# Genetic mechanisms of *Coxiella burnetii* lipopolysaccharide phase variation

**DOI:** 10.1371/journal.ppat.1006922

**Published:** 2018-02-26

**Authors:** Paul A. Beare, Brendan M. Jeffrey, Carrie M. Long, Craig M. Martens, Robert A. Heinzen

**Affiliations:** 1 Coxiella Pathogenesis Section, Laboratory of Bacteriology, Rocky Mountain Laboratories, National Institute of Allergy and Infectious Diseases, National Institutes of Health, Hamilton, Montana, United States of America; 2 Bioinformatics and Computational Biosciences Branch, Rocky Mountain Laboratories, National Institute of Allergy and Infectious Diseases, National Institutes of Health, Hamilton, Montana, United States of America; 3 Research Technologies Section, Rocky Mountain Laboratories, National Institute of Allergy and Infectious Diseases, National Institutes of Health, Hamilton, Montana, United States of America; Stanford University School of Medicine, UNITED STATES

## Abstract

*Coxiella burnetii* is an intracellular pathogen that causes human Q fever, a disease that normally presents as a severe flu-like illness. Due to high infectivity and disease severity, the pathogen is considered a risk group 3 organism. Full-length lipopolysaccharide (LPS) is required for full virulence and disease by *C*. *burnetii* and is the only virulence factor currently defined by infection of an immunocompetent animal. Transition of virulent phase I bacteria with smooth LPS, to avirulent phase II bacteria with rough LPS, occurs during in vitro passage. Semi-rough intermediate forms are also observed. Here, the genetic basis of LPS phase conversion was investigated to obtain a more complete understanding of *C*. *burnetii* pathogenesis. Whole genome sequencing of strains producing intermediate and/or phase II LPS identified several common mutations in predicted LPS biosynthesis genes. After passage in broth culture for 30 weeks, phase I strains from different genomic groups exhibited similar phase transition kinetics and elevation of mutations in LPS biosynthesis genes. Targeted mutagenesis and genetic complementation using a new *C*. *burnetii* nutritional selection system based on lysine auxotrophy confirmed that six of the mutated genes were necessary for production of phase I LPS. Disruption of two of these genes in a *C*. *burnetii* phase I strain resulted in production of phase II LPS, suggesting inhibition of the encoded enzymes could represent a new therapeutic strategy for treatment of Q fever. Additionally, targeted mutagenesis of genes encoding LPS biosynthesis enzymes can now be used to construct new phase II strains from different genomic groups for use in pathogen-host studies at a risk group 2 level.

## Author summary

*Coxiella burnetii* is the causative agent of Q fever, an acute febrile illness that can develop into a persistent focalized infection, such as endocarditis or vascular disease. Currently, the only licensed vaccine against Q fever is Q-Vax, a formalin-inactivated whole-cell preparation of the virulent *C*. *burnetii* Henzerling strain that is only available in Australia. Full-length (smooth) LPS is required for full virulence and efficacious Q fever vaccines. Indeed, various immune assays show LPS as an immunodominant antigen. Upon serial passage of *C*. *burnetii* in embryonated hen’s eggs, tissue culture, or synthetic medium, a smooth-to-rough (truncated) LPS transition occurs that results in avirulence. Using laboratory strains in various stages of phase variation, we defined several genetic pathways associated with LPS phase transition. In addition to defining genes responsible for production of a critical virulence factor, this study reveals LPS enzymes early in the biosynthetic pathway that can be subjected to small molecule screens to identify compounds that inhibit production of phase I LPS. The resulting loss of *C*. *burnetii* pathogenicity may aid immune clearance of the organism to alleviate human disease.

## Introduction

*Coxiella burnetii* is a gram-negative intracellular bacterial pathogen and the etiological agent of the zoonosis Q fever. Sheep, goats, and dairy cattle are considered relevant animal reservoirs for human infection. Infection generally results from inhalation of aerosols containing the highly infectious and stable bacterium that arise from contaminated animal products [1]. Q fever typically presents as an acute flu-like illness that often goes untreated. In rare occasions, Q fever manifests as a more serious persistent focalized infection (formerly termed chronic Q fever) that can result in endocarditis or vascular disease [2]. These infections require long-term treatment with doxycycline and hydroxychloroquine. While treatment significantly reduces mortality, it is not always effective, resulting in disease relapse [3–5]. Recent Q fever outbreaks in The Netherlands [6, 7] and U.S. [8] highlight Q fever as a public health threat.

*C*. *burnetii* undergoes a virulent-to-avirulent transition involving lipopolysaccharide (LPS) truncation known as phase variation [9]. The seminal work of Fiset and Stoker [10, 11] provided the conceptual framework for *C*. *burnetii* phase variation, a term originally coined to describe the peculiar serological behavior of *C*. *burnetii* strains. They demonstrated that organisms in phase II react with early (< 20 days) and late (>20 days) sera derived from infected guinea pigs, while organisms in phase I react only with late sera. The phenomenon was associated with culture history, i.e., natural isolates with phase I reactivity convert to phase II reactivity after approximately 10 passages in embryonated hen’s eggs. Fiset *et al*. [10] suggested that, although low passage *C*. *burnetii* produce both phase I and phase II antigens, phase II antigens are “masked” by phase I antigens and unavailable for antibody interactions in vitro. He further speculated that phase I and phase II antigens are biochemically different, with phase I and phase II antigens being “poor” and “good” antigens, respectively. These insightful propositions made over 60 years ago were validated upon showing that phase I antigen is LPS O-antigen [12–14] which sterically inhibits binding of antibodies directed against phase II antigens (surface proteins), deposition of complement, and recognition of toll-like receptor ligands, such as lipoproteins, from innate immune recognition by dendritic cells [12–17]. Accordingly, high passage and clonal phase II variants lack O-antigen altogether and are avirulent in a guinea pig infection model [12–14, 18, 19]. Biochemical analysis and genomic sequencing of the isogenic virulent Nine Mile phase I (NMI) (RSA493) and avirulent Nine Mile phase II (NMII) (RSA439) strains indicate LPS is the sole factor responsible for the disparate virulence of the two strains, and that O-antigen is the primary surface antigen recognized by phase I antiserum [14, 20, 21]. Indeed, fixed, whole-cell phase II bacteria generated by 20 egg passages of phase I bacteria are 100 to 300 times less effective as vaccines [22]. Thus, the only available vaccine for protection against Q fever (Q-Vax), is a formalin-inactivated preparation of whole cells of the virulent Henzerling phase I strain that is licensed for use in Australia [23]. Distinct genomic groups of *C*. *burnetii* produce structurally and antigenically different phase I LPS molecules [24]; however, cross protection is observed when vaccinated animals are challenged with heterologous phase I strains, indicating the presence of common epitopes [18, 22, 25]. The critical importance of full-length LPS in protective immunity induced by *C*. *burnetii* phase I vaccines suggests subunit vaccines based solely on protein antigens will be ineffective.

*C*. *burnetii* LPS phase variation is analogous to the smooth-to-rough LPS transition seen in the *Enterobacteriaceae*. *C*. *burnetii* phase variation results from gene mutation and is not to be confused with phase variation of other gram-negative bacteria, which is generally a reversible on-off regulatory process associated with production of several virulence factors, such as fimbriae, capsule, and LPS antigenicity [26]. *C*. *burnetii* LPS is the only virulence factor defined by infection of an immunocompetent animal [18]. Virulent organisms, producing full-length phase I (smooth) LPS and isolated from infections and natural sources, convert to avirulent organisms producing truncated phase II (rough) LPS upon serial passage in embryonated hen’s eggs, cell culture, or synthetic medium [11, 27–29]. The selective pressure promoting LPS transition is thought to involve energy conservation [30, 31]. The sugars comprising the inner/outer core regions and the repeating O-antigen subunits of LPS from the NMI strain have been identified [32–34]. Two unusual O-antigen sugars unique to *C*. *burnetii* LPS are virenose (6-deoxy-3-C-methylgulose) and dihydrohydroxystreptose (3-C-(hydroxymethyl)-L-lyxose) [32]. Unlike other gram-negative bacteria, where O-antigen generally has a defined repeat size [35], *C*. *burnetii* O-antigen has populations that differ in size and composition [24, 36]; consequently, its carbohydrate structure remains unresolved. A strain isolated from placental tissue of a guinea pig persistently infected with NMI produces an intermediate length LPS [37, 38]. This strain, termed Nine Mile Crazy (NMC) (RSA514), produces an LPS that weakly reacts with polyclonal anti-phase I LPS antiserum, a result that correlates with the absence of repeating O-antigen subunits and virenose [37, 39]. The LPS structure of NMII contains tetra-acylated lipid A linked to an inner core consisting of three 3-deoxy-D-manno-2-octulosonic acid (KDO) molecules, two terminal D-mannose molecules, and 2- and 3,4-linked D-*glycero*-D-*manno*-heptose molecules [33, 40–42]. The lipid A of NMI and NMII is identical and fails to signal through toll-like receptor (TLR) 2 or TLR4 [43]. Phase II LPS is missing both the outer core and repeating O-antigen sugars, including dihydrohydroxystreptose and virenose [44].

The genetic lesion(s) resulting in *C*. *burnetii* phase I to phase II transition are undefined. NMC and NMII contain large overlapping chromosomal deletions that eliminate *cbu0676* to *cbu0700* (31,570 bp) and *cbu0678* to *cbu698* (25,997 bp), respectively [45, 46]. This region contains genes implicated in virenose synthesis [47, 48]. The lack of virenose in the LPS of NMC and NMII agrees with this hypothesis [33, 39]. The large deletion of NMC accounts for an intermediate length LPS. However, the smaller deletion of NMII does not explain missing outer core and O-antigen sugars [49]. The large chromosomal deletion of NMII, avirulence in a guinea pig model of infection, and lack of reversion to phase I LPS are the basis for classifying NMII as a risk group 2 organism. Remaining *C*. *burnetii* strains are considered risk group 3 bacteria [18, 50, 51]. The precise genetic lesion(s) accounting for the rough LPS of NMII is unknown. Indeed, additional phase II strains lacking a large deletion have been characterized [52, 53]. LPS gene expression profiling did not identify mutations responsible for transition to phase II [52, 53].

In this study, we show that phase variation occurs in a similar fashion between *C*. *burnetii* of different genomic groups, resulting in common intermediate and phase II LPS forms. Site-directed mutagenesis of virulent *C*. *burnetii*, using a new method of genetic selection based on *C*. *burnetii* lysine auxotrophy, identified several mutations responsible for LPS phase transition. In particular, mutation of *cbu0533*, encoding a undecaprenyl-phosphate alpha-N-acetylglucosamine phosphotransferase, is the genetic lesion responsible for the phase II LPS of NMII. Collectively, our results reveal the genetic complexity of LPS modifications by *C*. *burnetii* that are directly related to virulence potential.

## Results

### Recognition of LPS forms using monoclonal antibodies

A distinguishing property of phase I and phase II *C*. *burnetii* is surface charge [18, 54]. The absence of O-antigen sugars in phase II strains is associated with hydrophobicity and spontaneous agglutination [18, 54]. These properties allow distinction from hydrophilic phase I bacteria during axenic growth. Phase II bacteria clump in liquid media and form opaque colonies on agarose plates with a defined border. Conversely, phase I bacteria disperse in media and form translucent colonies with an undefined border (S1 Fig). These growth phenotypes are useful scores for LPS phase transition in *C*. *burnetii*.

**Fig 1:**
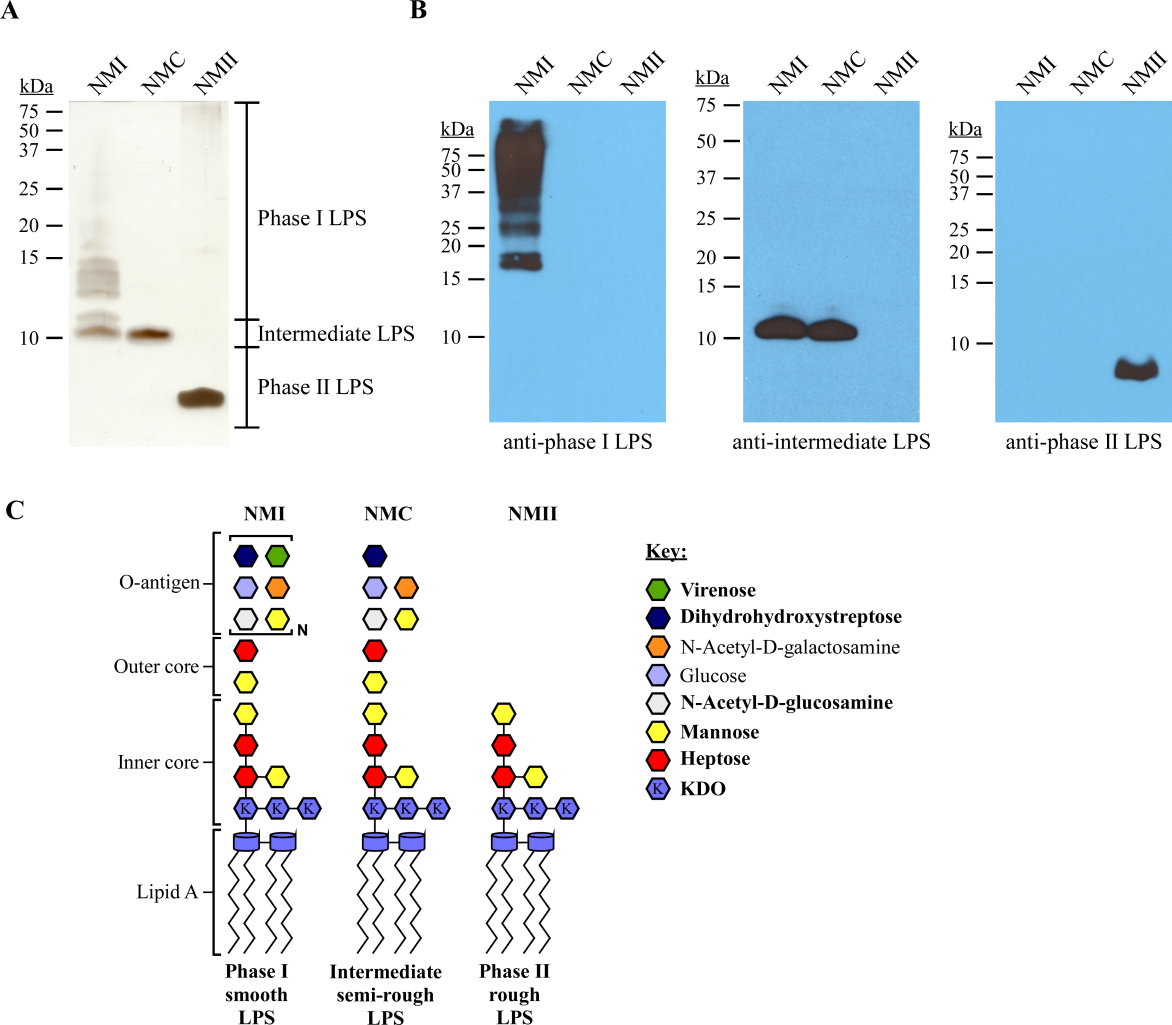
Antibody detection of *C*. *burnetii* LPS forms. LPS was extracted from *C*. *burnetii* NMI (RSA493), NMC (RSA514), and NMII (RSA439) following culture in ACCM-2 for 7 days. (A) LPS was separated by SDS-PAGE and silver stained, (B) LPS was separated by SDS-PAGE, blotted, and probed with antibodies specific to phase I, intermediate, or phase II LPS. NMI, NMC and NMII have unique LPS profiles consisting of multiple bands above 10 kDa, a single band of ~11 kDa and a single band at ~3 kDa, respectively. (C) Model of putative LPS structures of NMI and NMC is based on chemical composition and the previously determined LPS structure of NMII. Bonds between sugars of the outer core and O-antigen are not shown as the structure is unknown. Bold text in the key indicates highly abundant sugars. KDO is defined as 3-deoxy-D-manno-2-octulosonic acid.

NMI, NMC, and NMII are isogenic strains producing prototypical phase I, intermediate, and phase II LPS forms, respectively (Fig 1A) [52]. The specificity of monoclonal antibodies against LPS of NMI (anti-phase I LPS), NMC (anti-intermediate LPS) and NMII (anti-phase II LPS) were examined by immunoblot (Fig 1B). Anti-phase I LPS antibody reacted with O-antigen of full-length LPS as indicated by a laddering profile above 15 kDa not obvious by silver stain (Fig 1A). The anti-intermediate LPS antibody reacted with an intermediate size LPS of ~11 kDa in both NMI and NMC. The anti-phase II LPS antibody reacted with an ~3 kDa LPS specific to NMII [14]. Representative LPS structures are depicted in Fig 1C [31, 36, 39, 42, 44, 55]. These antibodies were used in remaining experiments to characterize LPS produced by wild type and mutant strains of *C*. *burnetii*.

### LPS phase transition is similar in *C*. *burnetii* strains

**Fig 2:**
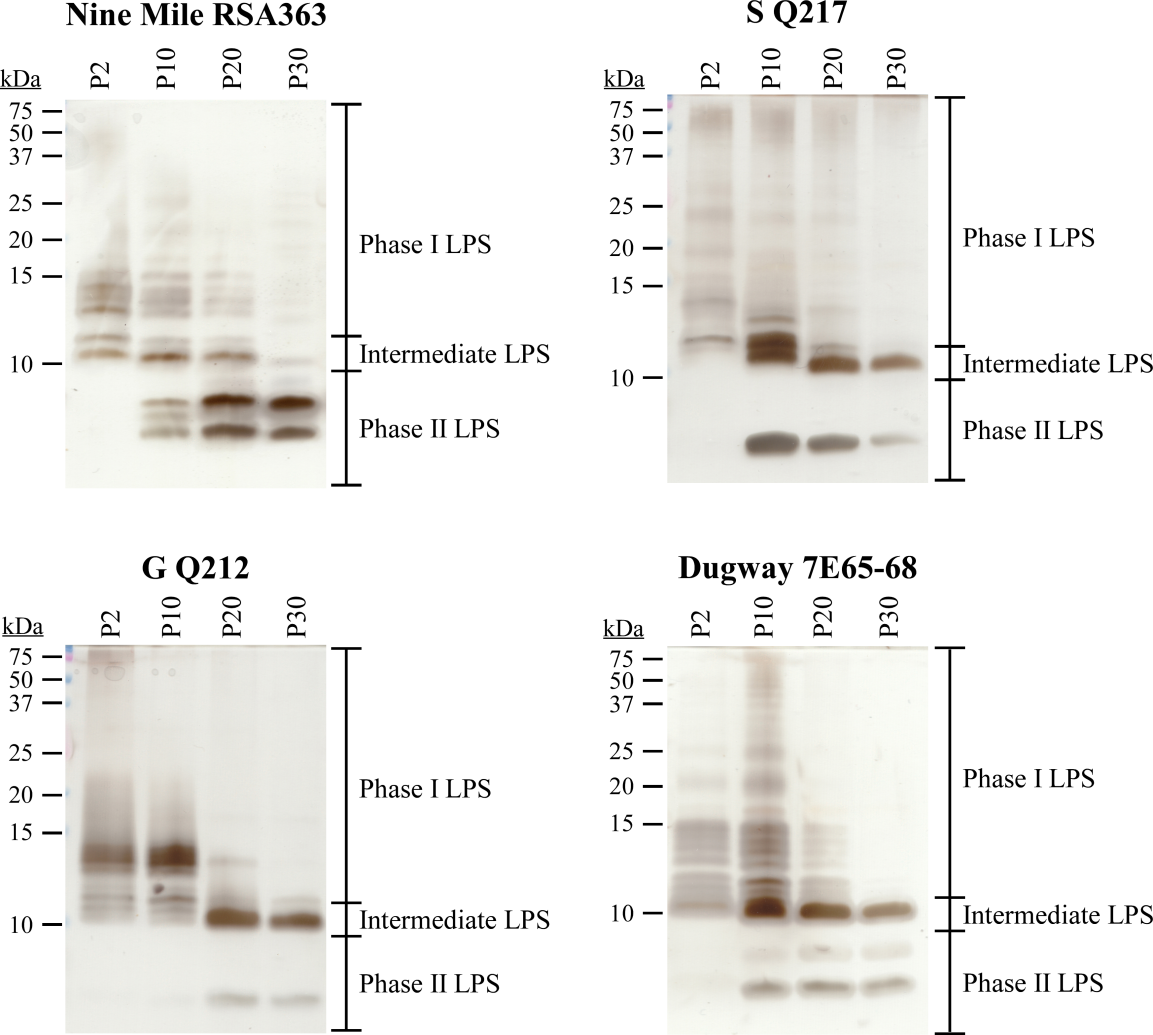
Phase transition of strains from different genomic groups following serial passage in axenic media. NMI (RSA363), S (Q217), G (Q212), and Dugway (7E65-68) were passaged weekly in ACCM-2 for 30 weeks. LPS was extracted at passage 2, 10, 20 and 30, separated by SDS-PAGE, and silver stained. Each strain has a phase I LPS profile at passage 2. Strains subject to additional passage produce increasing amounts of intermediate and phase II LPS.

*C*. *burnetii* phase variation is described as a non-reversible shift from full-length LPS of virulent phase I bacteria to truncated LPS of avirulent phase II bacteria [11, 56]. Ftacek *et al*. previously demonstrated that serial passage of the *C*. *burnetii* Priscilla phase I strain in embryonated hen’s eggs results in sequential transition from high-to-intermediate-to-low molecular weight LPS molecules [27]. To further examine LPS phase variation in *C*. *burnetii*, Nine Mile (RSA363), S (Q217), G (Q212), and Dugway (7E65-68) phase I strains were serially passaged weekly for 30 weeks in the synthetic medium acidified citrate cysteine medium-2 (ACCM-2), and LPS isolated at passage 2, 10, 20 and 30. These strains are isolated from disparate sources and fall within different genomic groups (S1 Table). Also included was NMC. LPS profiles following passage were examined by silver stain and immunoblot (Fig 2 and S2 Fig). At passage 2, the phase I LPS profile of each phase I strain was unique, indicating different forms of LPS exist within the *Coxiella* genus, as previously reported [14, 24, 33]. During subsequent passage, LPS profiles changed in a similar fashion with the appearance of intermediate (~11 kDa) and phase II (~3 kDa) LPS forms. The rate of change differed slightly among phase I strains. At passage 30, S (Q217), G (Q212), and Dugway (7E65-68) had similar intermediate and phase II LPS profiles. However, phase II LPS of S (Q217) appeared to decrease as passage number increased, suggesting reversion back to an intermediate LPS form. Also, intermediate LPS of Nine Mile (RSA363) decreased with a coincident increase in an upper phase II LPS form (~6 kDa). This same form was also faintly observed in Dugway (7E65-68) LPS starting at passage 10 (Fig 2). A previous study also showed an upper phase II LPS form following chick embryo passage of the Priscilla strain [27]. Passage of NMC resulted in an overall decrease in intermediate LPS and a subsequent increase in phase II LPS (S2A Fig). LPS of passaged strains was further examined by immunoblot (S2B Fig). Consistent with silver staining, phase I LPS (>15 kDa) decreased during passage with a coincident appearance of intermediate and phase II LPS. The ~6 kDa phase II LPS form was not recognized by anti-phase II antibody.

### Phase II LPS variants of *C*. *burnetii* have common chromosomal mutations

**Fig 3:**
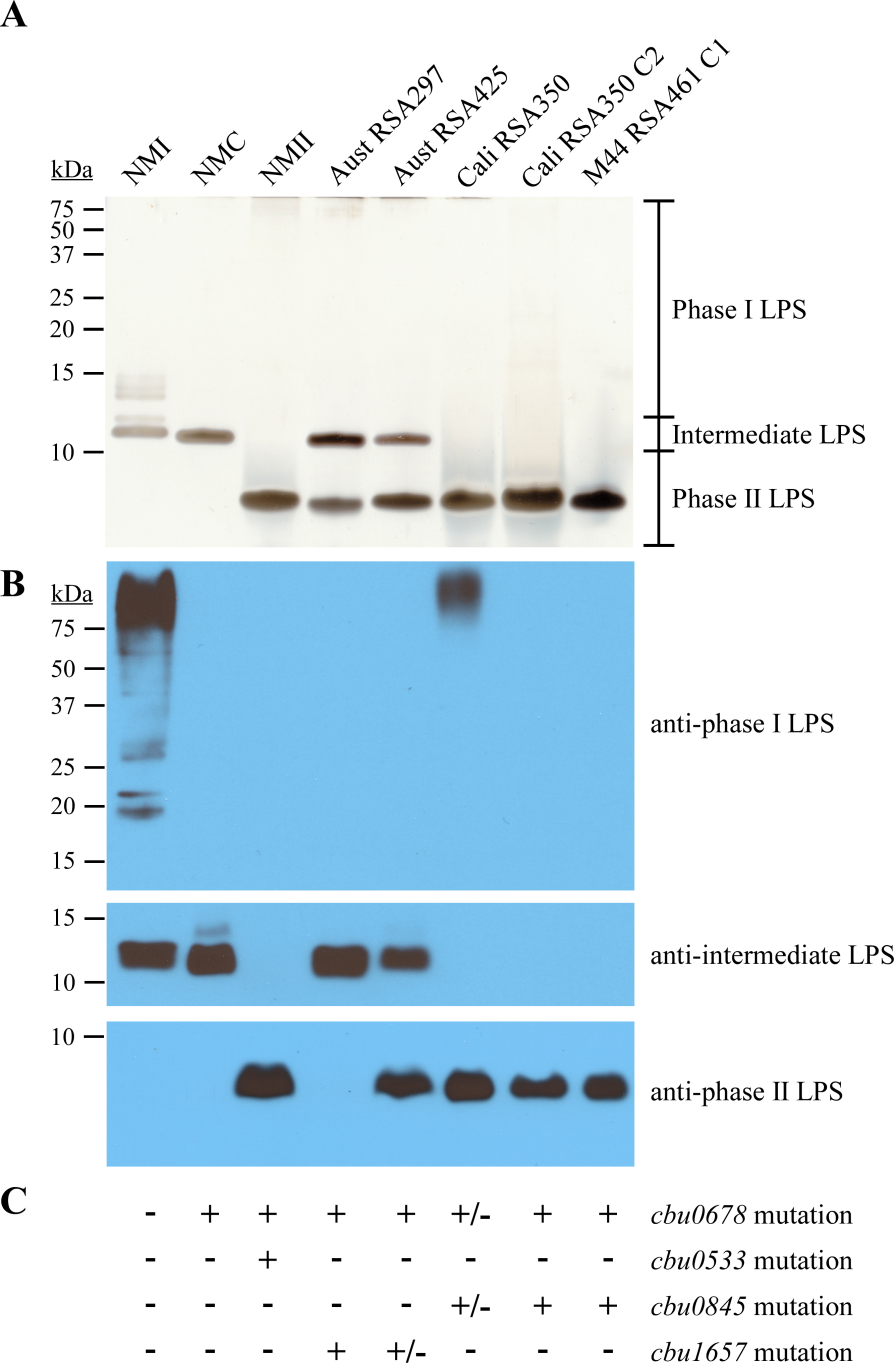
*C*. *burnetii* strains serologically in phase II have intermediate and/or phase II LPS. LPS was extracted from Australia (RSA297), Australia (RSA425), California 16 (RSA350), California 16 (RSA350) C2, and M44 (RSA461) C1, which are serologically in phase II [58], following culture in ACCM-2 for 7 days. LPS was separated by SDS-PAGE and compared to LPS from NMI, NMC, and NMII by (A) silver stain and (B) immunoblot using LPS-specific antibodies. Australia (RSA297) and Australia (RSA425) show an ~11 kDa intermediate and ~3 kDa phase II LPS. California 16 (RSA350), California 16 (RSA350) C2, and M44 (RSA461) C1 contain phase II LPS. A small amount of phase I LPS is observed only with the California 16 (RSA350) sample, indicating bacteria are producing phase I or phase II LPS. Despite a phase II band by silver stain, only intermediate LPS is detected in Australia (RSA297), suggesting the strain produces an antigenically distinct phase II LPS. Otherwise, banding patterns recapitulate those of silver stain gels. (C) The presence (+) or absence (-) of deleterious mutations within four LPS genes are indicated. Strains with a mixed population of an individual mutation are denoted as +/-.

Bacterial LPS transition from smooth-to-rough is usually associated with mutation of genes involved in LPS biosynthesis [30, 57]. To discover mutations associated with phase transition in *C*. *burnetii*, we characterized the LPS profiles and genomes of five strains previously serotyped as phase II [58] (Fig 3). The LPS profile of Australia (RSA297) and Australia (RSA425) were identical by silver stain, with each showing intermediate and phase II LPS (Fig 3A). Accordingly, immunoblot showed reactivity to anti-intermediate LPS antibody, but not to anti-phase I LPS antibody (Fig 3B). Interestingly, LPS of Australia (RSA297) did not react with anti-phase II LPS antibody, suggesting the Australia strains have different phase II LPS structures. The LPS of California (RSA350) mainly consisted of phase II LPS (~3 kDa) with a small amount of phase I LPS detectable only by immunoblot (Fig 3B). A clone of this strain derived by micromanipulation [59], California (RSA350) C2, contained only phase II LPS (Fig 3B). Similarly, M44 (RSA461) C1, a clonal strain obtained by plaque formation [60], displayed only phase II LPS (Fig 3A and 3B).

To define the genetic mutations associated with production of intermediate and phase II LPS, we conducted whole genome sequencing of the five phase II strains, in addition to NMC and NMII (Fig 3). The identified mutations are listed in S2 Table and include mutations in four predicted LPS biosynthesis genes: *cbu0678*, *cbu0533*, *cbu0845*, and *cbu1657* (Fig 3C). Mutation of *cbu0678* was common to all strains, including a complete deletion in NMC and a partial deletion in NMII. The *cbu0678* gene is located in a chromosomal region implicated in virenose biosynthesis [46–49]. A single mutation in *cbu0533* was specific to NMII. CBU0533 has homology to *E*. *coli* WecA (Rfe), which is a undecaprenyl-phosphate alpha-N-acetylglucosamine phosphotransferase that initiates O-antigen synthesis [61]. Two different disruptive mutations in *cbu0845* were present in California (RSA350) and M44 (RSA461) C1 strains. CBU0845 has homology to the GDP-mannose dehydrogenase family of LPS enzymes, which in *Pseudomonas aeruginosa*, is involved in O-antigen synthesis [62]. Mutated *cbu1657* was present in Australia (RSA297) and Australia (RSA425), although only ~28% of sequence reads in Australia RSA425 had this mutation. CBU1657 has homology to an alpha-L-*glycero*-D-*manno*-heptose beta-1,4-glucosyltransferase, which in *Klebsiella pneumoniae*, transfers glucose to heptose I in the inner core [63].

### CBU0678 is essential for production of phase I LPS

**Fig 4:**
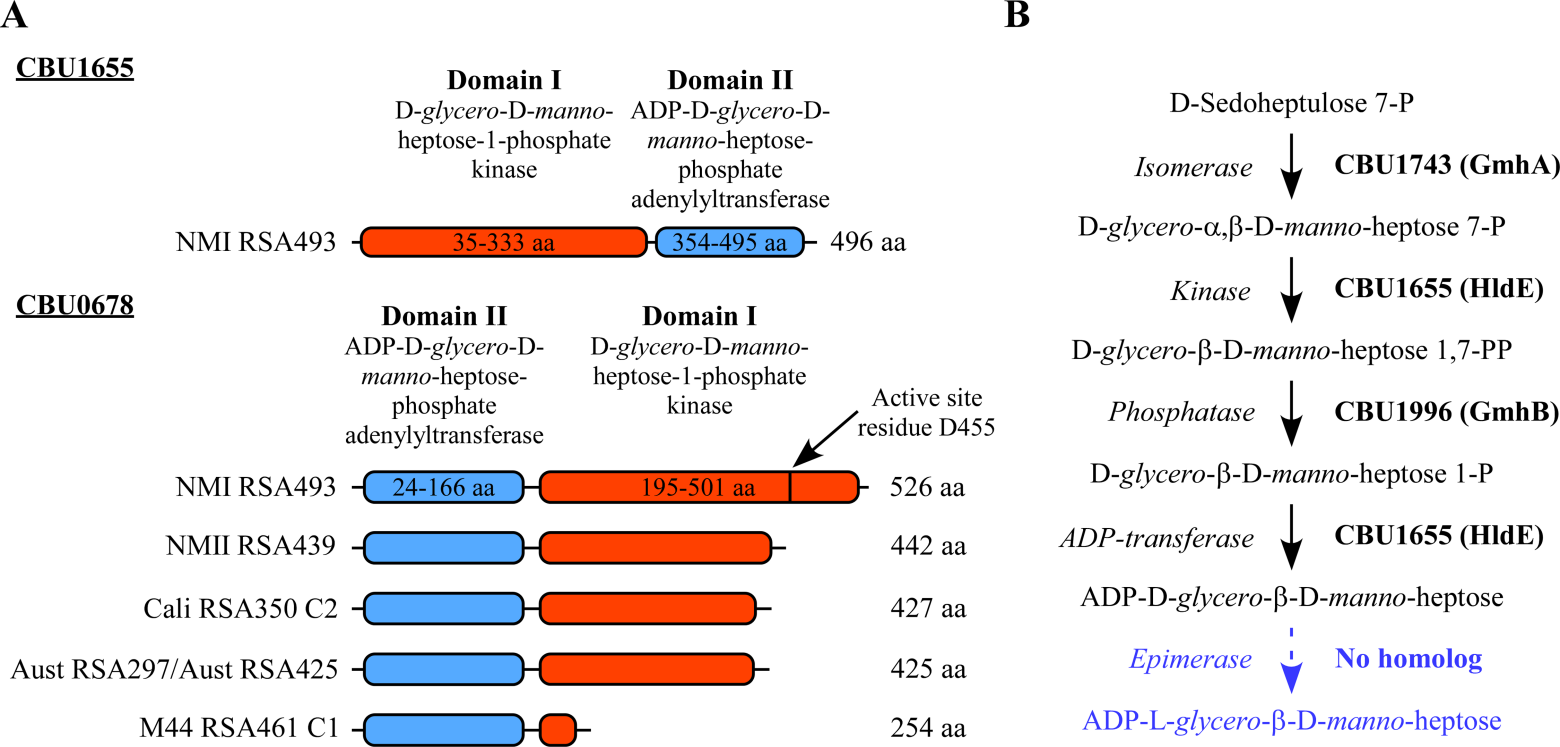
Domain structure of CBU1655 and CBU0678. (A) Schematic of the domain structure of the NMI CBU1655, a predicted D-*glycero*-D-*manno*-heptose-7-phosphate 1-kinase/D-*glycero*-D-*manno*-heptose-1-phosphate adenylyltransferase. Also shown is the domain structure of CBU0678 from phase II strains compared to that of NMI (RSA493). The location of the predicted active site residue, aspartate 455 (D455), is shown. CBU1655 is full-length in all strains. CBU0678 has a reversed domain structure compared to CBU1655. All depicted phase II strains contain a frameshift mutation in *cbu0678* that results in a truncated protein. (B) Schematic for the putative ADP-D-*glycero*-β-D-*manno*-heptose synthesis pathway in *C*. *burnetii*. *C*. *burnetii* genes predicted to be involved at each step are printed in bold. The last step is displayed in blue as *C*. *burnetii* LPS does not contain ADP-L-*glycero*-β-D-*manno*-heptose.

The mutation of *cbu0678* in all sequenced strains suggested the gene is required for transition of intermediate to phase I LPS. CBU0678 has homology to CBU1655, which is annotated as a bifunctional sugar kinase/adenylyltransferase containing two domains (Fig 4A). Domain I of CBU1655 is a D-*glycero*-D-*manno*-heptose-7-phosphate 1-kinase while domain II is a D-*glycero*-D-*manno*-heptose-1-phosphate adenylyltransferase. Based on homology to *E*. *coli* HldE, CBU1655 is predicted to synthesize ADP-D-*glycero*-β-D-*manno*-heptose [64], a sugar found in the *C*. *burnetii* inner core [42] (Fig 4B). CBU0678 has an unconventional, reversed domain arrangement (Fig 4A). All *cbu0678* mutations result in a truncated C-terminus that eliminates the predicted active site of domain I [64] (Fig 4A).

**Fig 5:**
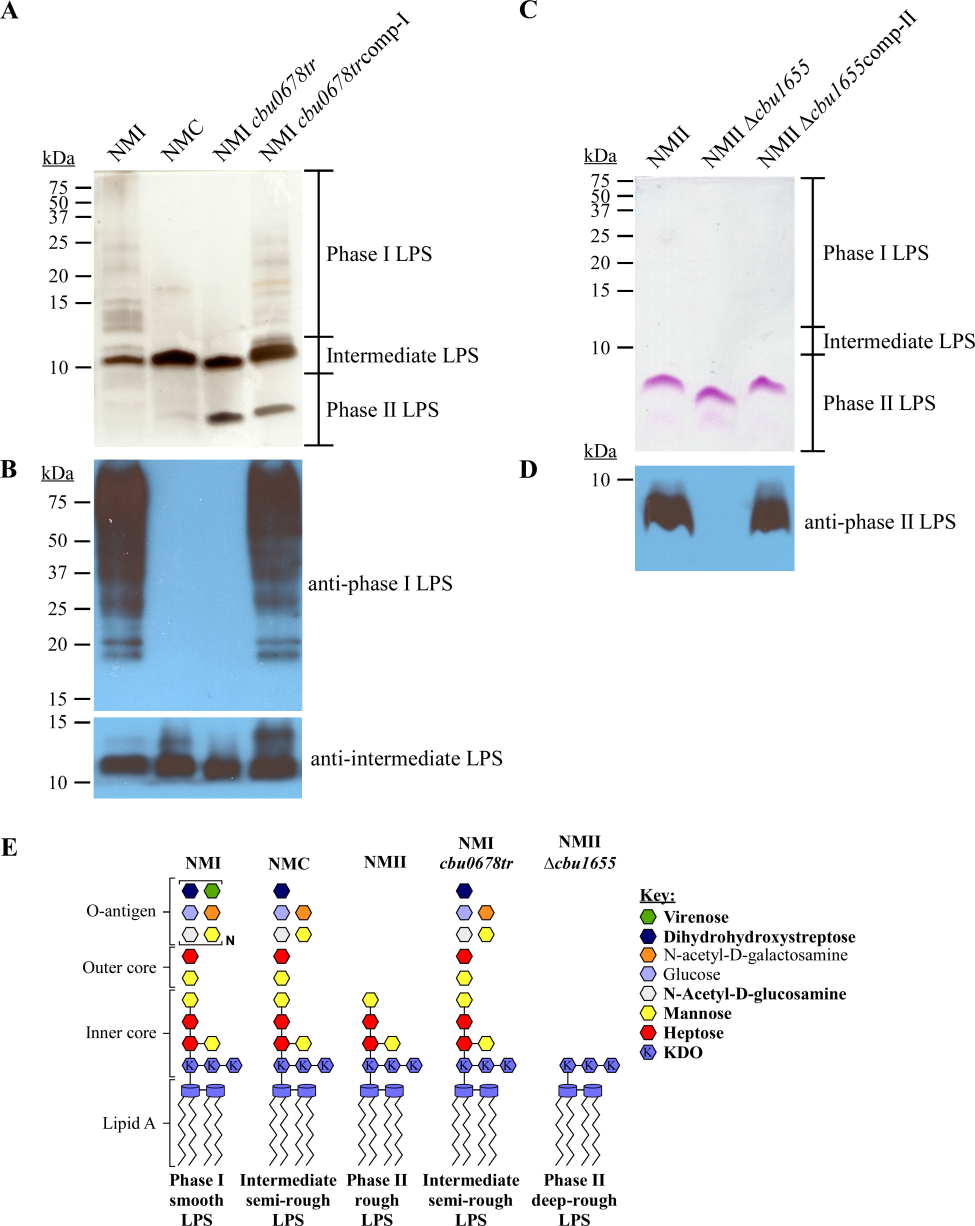
CBU0678 is essential for production of phase I LPS. LPS was extracted from NMI, NMC, NMI *cbu0678tr*, or NMI *cbu0678tr*comp-I following culture in ACCM-2 for 7 days. LPS was separated by SDS-PAGE then analyzed by (A) silver stain or (B) immunoblot probed with LPS-specific antibodies. NMI *cbu0678tr* produces intermediate and phase II LPS, but not phase I LPS. Expression of wild type *cbu0678* in NMI *cbu0678tr* restores production of phase I LPS, indicating CBU0678 is essential for synthesis of phase I LPS. LPS from NMII, NMII Δ*cbu1655*, and NMII Δ*cbu1655*comp-II was isolated using a modified microextraction protocol following culture in ACCM-2 for 7 days. LPS was separated by SDS-PAGE and visualized by (C) glycoprotein staining or (D) immunoblot probed with anti-phase II LPS antibody. NMII Δ*cbu1655* produces a deep-rough phase II LPS (< 3 kDa), smaller than that of NMII, which is not recognized by anti-phase II LPS antibody. Expression of wild type *cbu1655* in NMII Δ*cbu1655* restores production of typical phase II LPS and antibody recognition. These data are consistent with a predicted role of CBU1655 in producing the first heptose of the *C*. *burnetii* inner core, which is a component of the epitope recognized by anti-phase II LPS antibody. (E) Model of putative LPS structures of NMI *cbu0678tr* and NMII Δ*cbu1655* compared to those of NMI, NMC, and NMII. Bold text in the key indicates highly abundant sugars. KDO is defined as 3-deoxy-D-manno-2-octulosonic acid.

To define the roles of CBU0678 and CBU1655 in LPS biosynthesis, the encoding genes were mutated in NMI and NMII, respectively. The *cbu0678* mutant (*cbu0678tr*) was engineered to express a truncated version of the protein containing amino acids (AA) 1 to 437. This generates the mutation observed in NMII [46] (Fig 4A). NMI *cbu0678tr* contained intermediate and phase II LPS, but not phase I LPS (Fig 5A and 5B). Expression of a wild type copy of *cbu0678* in NMI *cbu0678tr* rescued production of phase I LPS (Fig 5A and 5B). These data indicate that *cbu0678* is essential for production of phase I LPS and that disruption of the enzyme results in an intermediate length LPS. The small amount of observed phase II LPS is a consequence of passage in synthetic medium, which is required to create and clone the mutant. The predicted function of CBU1655 is synthesis of the first heptose of the inner core of *C*. *burnetii* LPS. To test this idea, *cbu1655* was deleted in NMII. Isolation of LPS from NMII Δ*cbu1655* using a traditional hot phenol method was unsuccessful, a result possibly explained by a less water-soluble form of LPS lacking heptose and mannose [14]. Therefore, a modified LPS extraction method was used [65]. NMII Δ*cbu1655* produced a deep rough phase II LPS that was smaller than NMII (Fig 5C) that did not react with anti-phase II LPS antibody (Fig 5D). These data are consistent with CBU1655-directed synthesis of the first heptose in *C*. *burnetii* LPS. Expression of *cbu1655* in the mutant rescued production of NMII phase II LPS, confirming *cbu1655* involvement in inner core biosynthesis. The predicted LPS structures of *cbu0678tr* and Δ*cbu1655* are depicted in Fig 5E.

### A single amino acid deletion in CBU0533 causes phase II LPS of NMII

**Fig 6:**
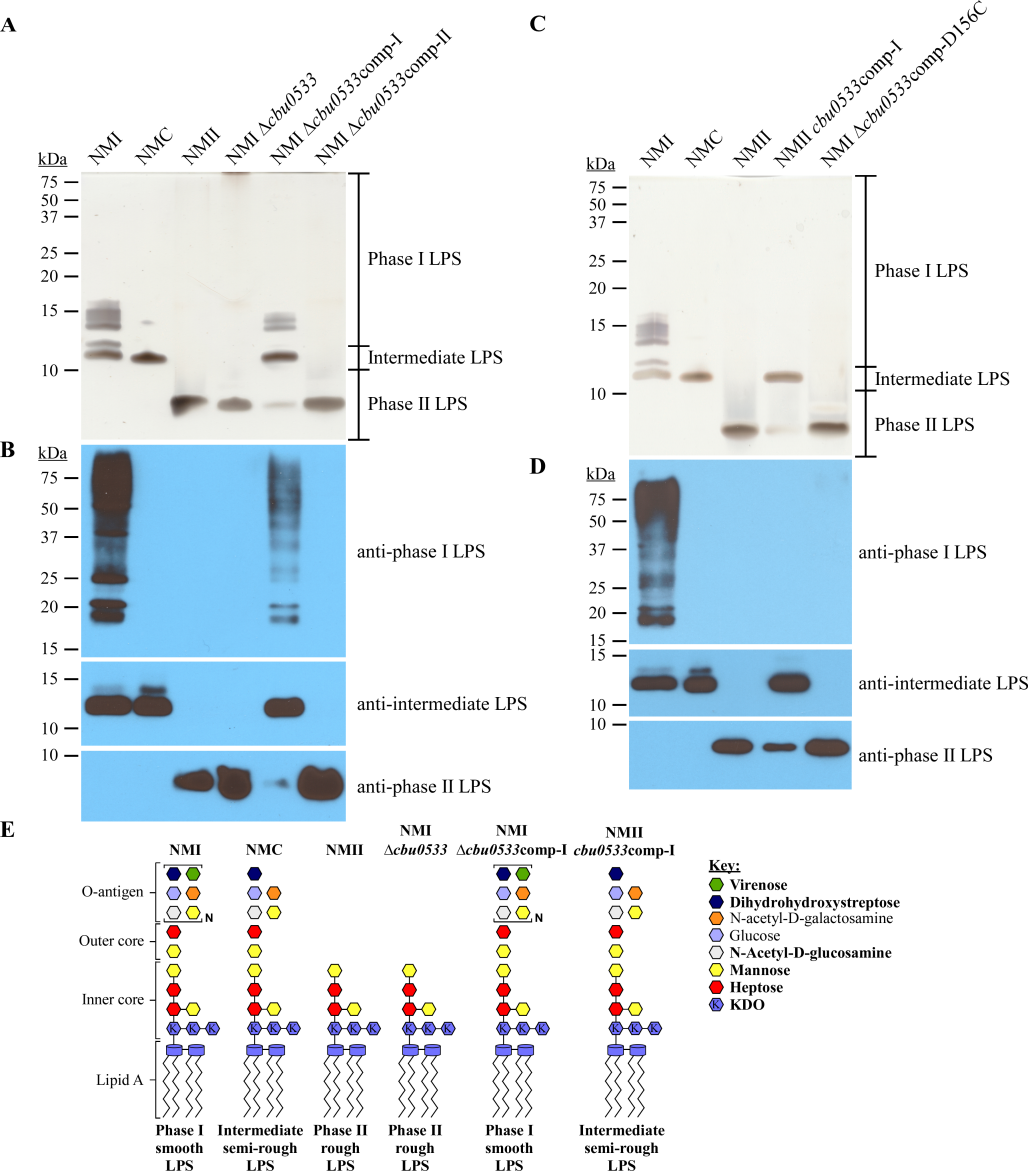
Mutation of *cbu0533* results in phase II LPS of NMII. LPS was extracted from NMI Δ*cbu0533*, NMI Δ*cbu0533*comp-I, and NMI Δ*cbu0533*comp-II following culture in ACCM-D for 7 days. LPS was separated by SDS-PAGE and compared to LPS from NMI, NMC, and NMII by (A) silver stain and (B) immunoblot using LPS-specific antibodies. NMI Δ*cbu0533* produces a phase II LPS similar to that of NMII. Expression of a wild type NMI copy (comp-I) of *cbu0533*, but not a NMII copy (comp-II) of *cbu0533*, in NMI Δ*cbu0533* restores production of phase I LPS. LPS was extracted from NMII *cbu0533*comp-I and NMI Δ*cbu0533*comp-D156C following culture in ACCM-D for 7 days. Samples were compared to LPS from NMI, NMC, and NMII. LPS was separated by SDS-PAGE and compared to LPS from NMI, NMC, and NMII by (C) silver stain and (D) immunoblot using LPS-specific antibodies. Expression of wild type *cbu0533* in NMII results in production of intermediate LPS, consistent with the presence of a large deletion in NMII preventing restoration back to phase I LPS. Expression of the active site mutant *cbu0533*-D156C in NMI Δ*cbu0533* does not restore synthesis of phase I LPS. These data demonstrate that mutation of *cbu0533* causes production of phase II LPS in NMII. (E) Model of putative LPS structures of NMI Δ*cbu0533*, NMI Δ*cbu0533*comp-I and NMII *cbu0533*comp-I compared to those of NMI, NMC, and NMII. Bold text in the key indicates highly abundant sugars. KDO is defined as 3-deoxy-D-manno-2-octulosonic acid.

CBU0533 is a predicted undecaprenyl-phosphate alpha-N-acetylglucosamine phosphotransferase. In *E*. *coli*, the enzyme is responsible for initiation of O-antigen elongation [66]. NMII *cbu0533* contains a 3 bp in-frame deletion that results in elimination of the first leucine (AA 168) in a string of five leucine residues (S3 Fig). This leucine motif is predicted to comprise a membrane-spanning region of the protein. To examine the role of *cbu0533* in LPS biosynthesis, *cbu0533* was deleted in NMI using a newly developed nutritional selection system based on lysine auxotrophy of *C*. *burnetii* [67]. Rescue of *C*. *burnetii* growth in ACCM-D media lacking lysine is accomplished by expression of *lysCA* (*lpp1774*) from *Legionella pneumophila* (S4 Fig). The LPS profile of NMI Δ*cbu0533* was identical to that of NMII (Fig 6A and 6B). Complementation here and elsewhere employed gDNA from wild-type NMI (comp-I) or NMII (comp-II). Expression of a wild-type copy of *cbu0533* (comp-I) in NMI Δ*cbu0533* rescued production of phase I LPS. However, expression of *cbu0533*, containing the 3 bp in-frame deletion (comp-II), did not rescue (Fig 6A and 6B), suggesting the missing leucine residue of NMII CBU0533 is essential for function. To further examine the importance of leucine 168, we expressed NMI *cbu0533* in NMII. Expression resulted in the production of an intermediate LPS (Fig 6C and 6D). No phase I LPS was seen. This result was expected as full complementation of NMII with one gene to produce phase I LPS is not possible due to the large chromosomal deletion (*cbu0678* to *cbu0698*) of the strain [45, 46]. Indeed, as demonstrated in Fig 5, disruption of *cbu0678* alone results in production of an intermediate LPS. The leucine residue deleted in NMII CBU0533 is directly downstream of the predicted active site of WecA (S3 Fig) [68], suggesting the change may influence enzyme function. The active site of *E*. *coli* WecA has two aspartate residues, D156 and D159, (S3 Fig) that are conserved in *C*. *burnetii* [61]. To examine if D156 is critical for CBU0533 function, we expressed a mutant of *cbu0533* in NMI Δ*cbu0533* where D156 is replaced with a cysteine residue (*cbu0533*-D156C). The *cbu0533*-D156C construct did not complement NMI Δ*cbu0533* (Fig 6C and 6D), confirming D156 is necessary for function. The predicted structures of NMI Δ*cbu0533*, NMI Δ*cbu0533*comp-I, and NMII *cbu0533*comp-I are depicted in Fig 6E. Together, these results indicate that disruption of CBU0533 results in the severely truncated phase II LPS of NMII.

### Mutations in *cbu0845* also result in phase II LPS

**Fig 7:**
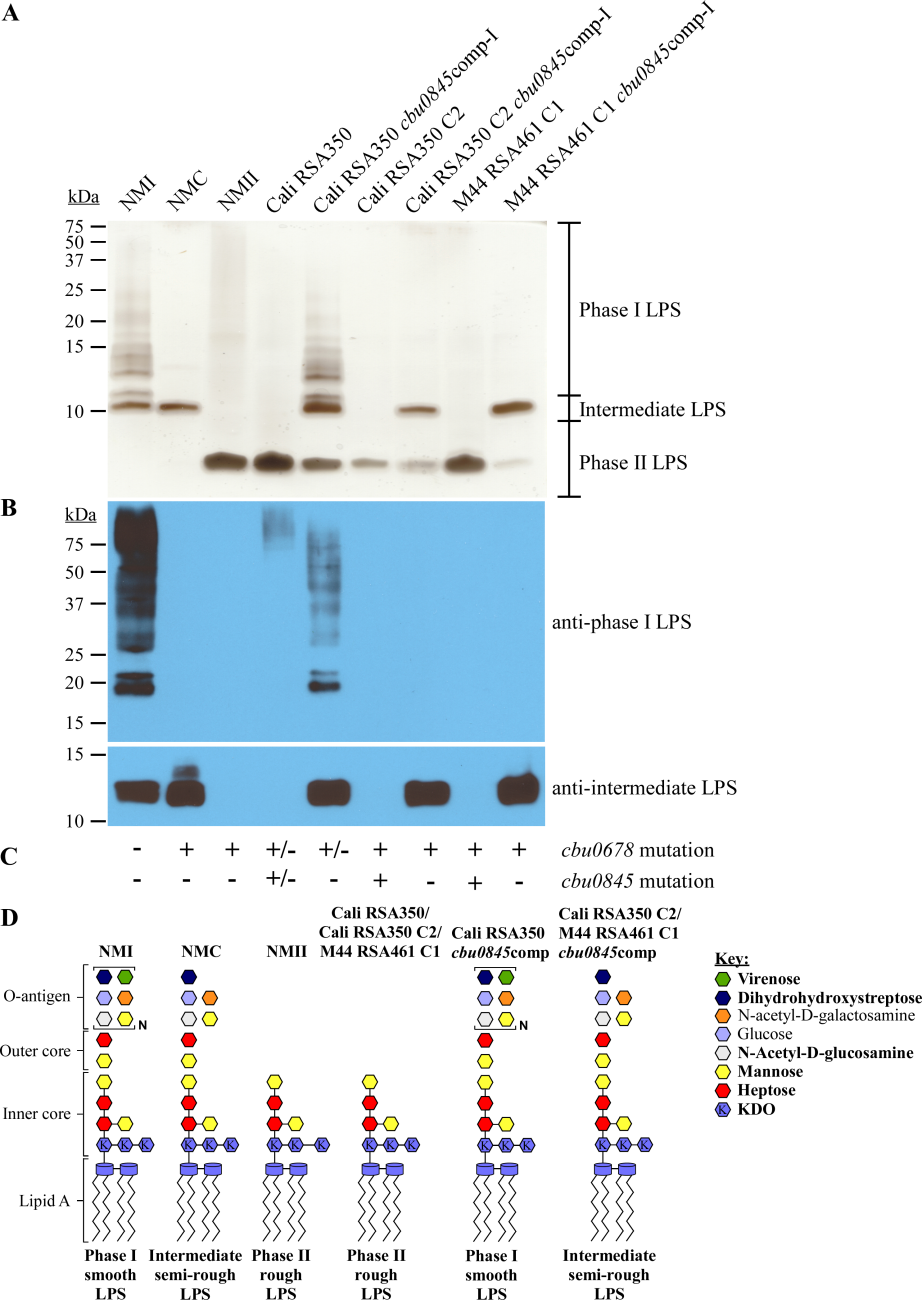
Mutations in *cbu0845* result in production of phase II LPS. LPS was extracted from California 16 (RSA350), California 16 (RSA350) *cbu0845*comp, California 16 (RSA350) C2, California 16 (RSA350) C2 *cbu0845*comp, M44 (RSA461) C1, and M44 (RSA461) C1 *cbu0845*comp following culture in ACCM-2 for 7 days. LPS was separated by SDS-PAGE and compared to LPS from NMI, NMC, and NMII by (A) silver stain and (B) immunoblot using LPS-specific antibodies. Expression of *cbu0845* in California 16 (RSA350) C2 and M44 (RSA461) C1 results in production of intermediate LPS due to the presence of a secondary mutation in *cbu0678*. Expression of *cbu0845* in California 16 (RSA350), which has bacteria with or without the *cbu0678* mutation, results in increased production of phase I LPS. (C) The presence (+) or absence (-) of deleterious mutations in *cbu0678* and *cbu0845* are indicated. Strains that have bacteria with or without gene mutations are denoted +/-. These data indicate that mutation of *cbu0845* results in production of phase II LPS. (D) Model of putative LPS structures of California 16 (RSA350), California 16 (RSA350) C2, M44 (RSA461) C1, California 16 (RSA350) *cbu0845*comp, California 16 (RSA350) C2 *cbu0845*comp, and M44 (RSA461) C1 *cbu0845*comp compared to those of NMI, NMC, and NMII. Bold text in the key indicates highly abundant sugars. KDO is defined as 3-deoxy-D-manno-2-octulosonic acid.

Domain analysis of CBU0845 suggested the enzyme is a GDP-mannose dehydrogenase. Phase II LPS profiles of California (RSA350), California (RSA350) C2, and M44 (RSA461) C1 correlated with mutation of *cbu0845*, but not *cbu0533* (Fig 3). Thus, CBU0845 likely directs synthesis of mannose in the *C*. *burnetii* outer core [41], the absence of which results in phase II LPS. Two mutations in *cbu0845* were identified by whole genome sequencing (S2 Table). Ninety-three percent of *cbu0845* sequence reads from California (RSA350) contained a single base pair deletion that causes a frameshift at amino acid 257 (S5 Fig). M44 (RSA461) C1 is clonal for a 24 base pair deletion that eliminates AA 280 to 287 (S5 Fig). Both strains also have mutations in *cbu0678*, necessary for intermediate-to-phase I transition, that cause a frameshift. However, in the case of California (RSA350), the mutation was found in only 19% of the sequence reads. Consistent with the presence of ~7% wild type *cbu0845* in California (RSA350), a small amount of phase I LPS was detected by immunoblot that was undetectable by silver stain (Fig 7A, 7B and 7C). Expression of wild-type *cbu0845* restored production of phase I LPS. Expression of wild type *cbu0845* in California (RSA350) C2, which is clonal for mutations in *cbu0845* and *cbu0678* (Fig 7C), resulted in production of intermediate LPS, but not phase I LPS, consistent with the *cbu0678* mutation preventing production of phase I LPS. Expression of wild type *cbu0845* in M44 (RSA461) C1, also clonal for mutations in *cbu0845* and *cbu0678* (Fig 7C), resulted in production of an intermediate LPS. A model for the predicted LPS structures of these strains is depicted in Fig 7D. These results confirm that mutation of *cbu0845* results in generation of phase II LPS. Moreover, they show a potential two-step transition to phase II LPS.

### Mutation of *cbu1657* generates a novel phase II LPS species

**Fig 8:**
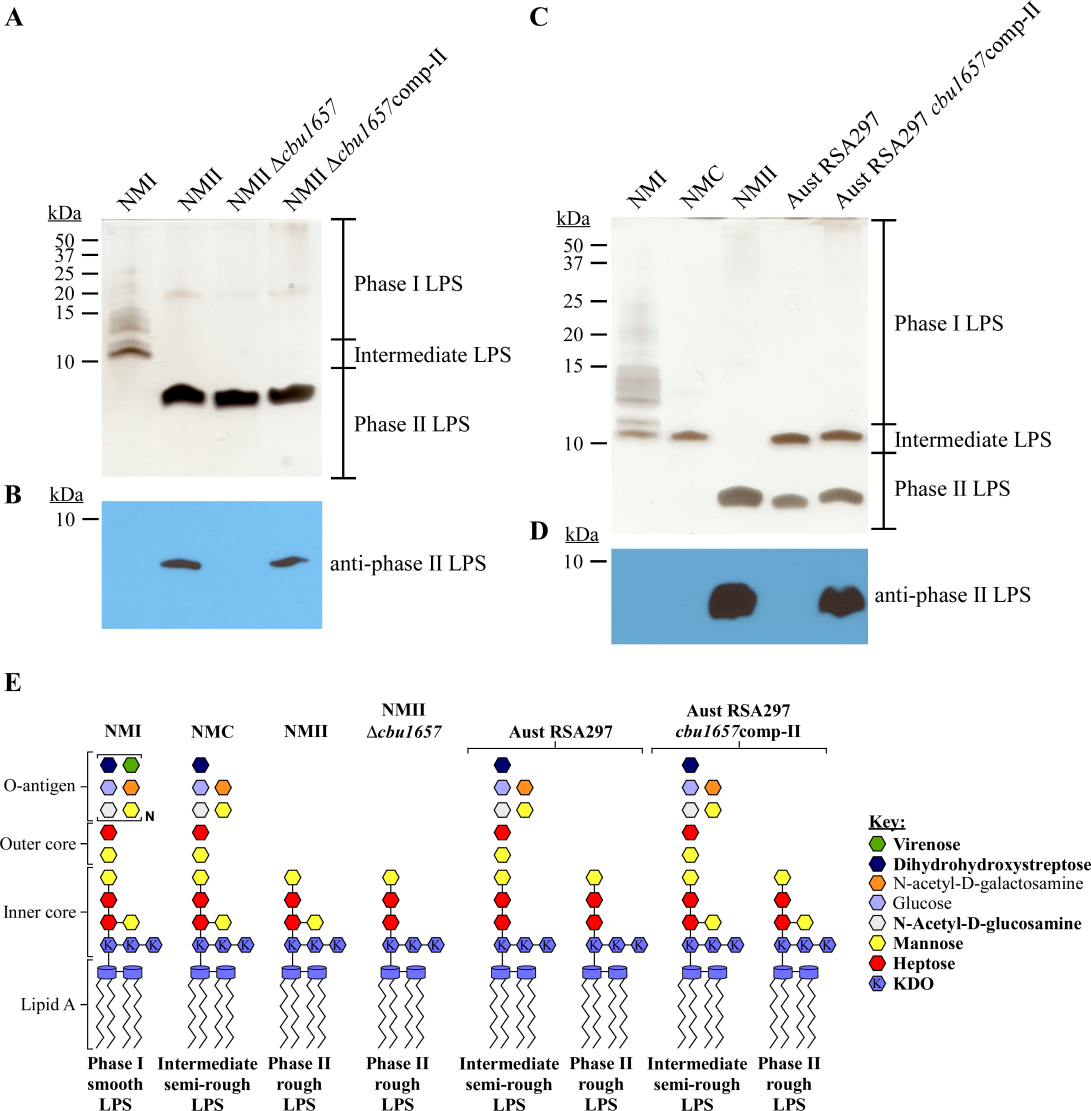
Mutation of *cbu1657* in Australia (RSA297) results in truncation of phase II LPS. LPS was extracted from NMII Δ*cbu1657* and NMII Δ*cbu1657*comp-II following culture in ACCM-2 for 7 days. LPS was separated by SDS-PAGE and compared to LPS from NMI and NMII by (A) silver stain and (B) immunoblot probed with anti-phase II LPS antibody. NMII Δ*cbu1657* produces a smaller phase II LPS than NMII that does not react with anti-phase II LPS antibody. Expression of wild type *cbu1657* in NMII Δ*cbu1657* restores phase II reactivity. LPS was extracted from Australia (RSA297) and Australia (RSA297) *cbu1657*comp-II following culture in ACCM-2 for 7 days. LPS was separated by SDS-PAGE and compared to LPS from NMI, NMC, and NMII by (C) silver stain or (D) immunoblot probed with anti-phase II LPS antibody. Expression of wild type *cbu1657* in Australia (RSA297) restores production of phase II LPS and anti-phase II LPS antibody reactivity. These data confirm that mutation of *cbu1657* results in truncation of phase II LPS in Australia (RSA297). (E) Model of putative LPS structures of NMII Δ*cbu1657*, Australia (RSA297), and Australia (RSA297) *cbu1675*comp-II compared to those of NMI, NMC, and NMII. Bold text in the key indicates highly abundant sugars. KDO is defined as 3-deoxy-D-manno-2-octulosonic acid.

As shown in Fig 3, LPS from the Australia (RSA297) strain produces a phase II LPS that does not react with antibody generated against NMII LPS. Sequencing of Australia (RSA425) and Australia (RSA297) identified a single base pair deletion in *cbu1657* that results in a frameshift (S2 Table). CBU1657 has homology to alpha-L-*glycero*-D-*manno*-heptose beta-1,4-glucosyltransferase. However, *C*. *burnetii* LPS contains D-*glycero*-D-*manno*-heptose, and not L-*glycero*-D-*manno*-heptose [41]. Based on the known structure of NMII phase II LPS [41, 42], we predicted that CBU1657 adds mannose to the first heptose of phase II LPS. This hypothesis is based on mannose having a 1,4 linkage to heptose I in phase II LPS and that *cbu1657* is predicted to encode a beta-1,4-glucosyltransferase [42, 59]. NMII Δ*cbu1657* produced an LPS slightly smaller than NMII that was not recognized by anti-phase II LPS antibody (Fig 8A and 8B). Reactivity was restored by expression of wild type *cbu1657*. Expression of *cbu1657* in Australia (RSA297) restored reactivity to anti-phase II LPS antibody (Fig 8C and 8D). These results show that mutation of *cbu1657* results in an antigenically-distinct phase II LPS that is smaller than NMII LPS, and that the 1,4-linked mannose is a necessary component of the epitope recognized by anti-phase II LPS antibody. Models of the LPS structures produced by the strains examined above are depicted in Fig 8E.

### Common pathways are targets in *C*. *burnetii* LPS phase transition

Experiments thus far utilized strains serologically in phase II to find mutations related to LPS transition. Ftacek *et al*, (2000) showed that serial passage of the Priscilla strain of *C*. *burnetii* in embryonated hen’s eggs results in loss of phase I LPS and accumulation of intermediate and phase II LPS forms [27]. Consistent with this finding, we demonstrated 30 passes of five *C*. *burnetii* strains in axenic media results in similar LPS changes (Fig 2 and S2 Fig). To examine global mutational changes associated with LPS transition, DNA was isolated from NMI (RSA363), S (Q217), G (Q212), Dugway (7E65-68), and NMC after 2, 10, 20 and 30 passes and sequenced. Fourteen mutations in 11 predicted LPS biosynthesis genes were identified (Table 1). In general, the frequency of mutation correlated with passage number. For example, NMI (RSA363) contained a 4 bp insertion in *cbu0839* resulting in a frameshift. This mutation increased in frequency to 30.4% of sequence reads at passage 10 and corresponded with the appearance of phase II LPS forms of ~3 and 6 kDa (Fig 2). Mutations in non-LPS biosynthetic genes were also identified, but their frequency did not increase with passage.

**Table 1:** Accumulation of mutations in *C*. *burnetii* LPS genes following long-term serial passage.

						Frequency of mutation (% reads)			
Gene	NMI homologue	Function	DNA change	Reference location^a^	AA change	P2	P10	P20	P30
**NMI RSA363**									
CBU0839	CBU0839	Glycosyltransferase	T -> TTTTA	791565	Frameshift	6.0	30.4	83.6	55.7
**NM Crazy RSA514**									
BMW92_RS02770	CBU0533	Undecaprenyl-phosphate alpha-N-acetylglucosaminephosphotransferase	CTAT -> C	478159–478161	5(L) -> 4(L)	0.6	31.9	67.2	68.4
BMW92_RS04285	CBU0841	alpha-D-QuiNAc alpha-1,3-galactosyltransferase	A(9) -> A(8)	794525	Frameshift	0.7	1.4	0.4	16.3
**S Q217**									
CBUG_RS07420	CBU0533	Undecaprenyl-phosphate alpha-N-acetylglucosaminephosphotransferase	C -> T	478069	T -> M	0.6	44.0	16.9	0.6
CBUG_RS06730	CBU0677	NAD dependent epimerase/dehydratase family	CAA -> C	619095–619096	Frameshift	0.0	5.2	50.0	73.6
CBUG_RS06700	CBU0683	Hypothetical protein	A -> AT	624743	Frameshift	0.0	33.6	16.3	0.0
CBUG_RS05880	CBU0846	UDP-glucose 6-dehydrogenase	G -> A	800651	E -> K	0.0	0.0	0.0	16.3
**G Q212**									
CBUG_RS06725	CBU0678	D-glycero-D-manno-heptose-1-phosphate adenylyltransferase / D-glycero-D-manno-heptose-7-phosphate 1-kinase	C -> G	619576	P -> A	0.0	7.0	65.4	39.0
CBUG_RS06725	CBU0678	D-glycero-D-manno-heptose-1-phosphate adenylyltransferase / D-glycero-D-manno-heptose-7-phosphate 1-kinase	G -> A	620461	G -> R	0.6	2.4	28.1	45.7
CBUG_RS10070	CBU1978	LPS-assembly protein LptD	C -> A	1890643	P -> T	0.0	0.0	7.7	37.9
**Dugway 7E65-68**									
CBUD_RS03460	CBU0674	Phosphoheptose isomerase	126 bp deletion	615934–616060	42 AA deletion	0.0	33.3	3.0	35.6
CBUD_RS03470	CBU0676	UDP-glucose 4-epimerase	C -> T	617404	Truncation	2.5	1.7	65.1	10.8
CBUD_RS04520	CBU0839	Glycosyltransferase	C -> T	791371	A -> V	0.0	93.2	12.9	71.5
CBUD_RS00875	CBU1947	Glucosamine-1-phosphate acetyltransferase / UDP-N-acetylglucosamine pyrophosphorylase	T -> C	1865375	L->P	5.4	8.0	8.5	12.5

^a^Nucleotide location of polymorphism relative to the Nine Mile RSA493 genome.

**Fig 9:**
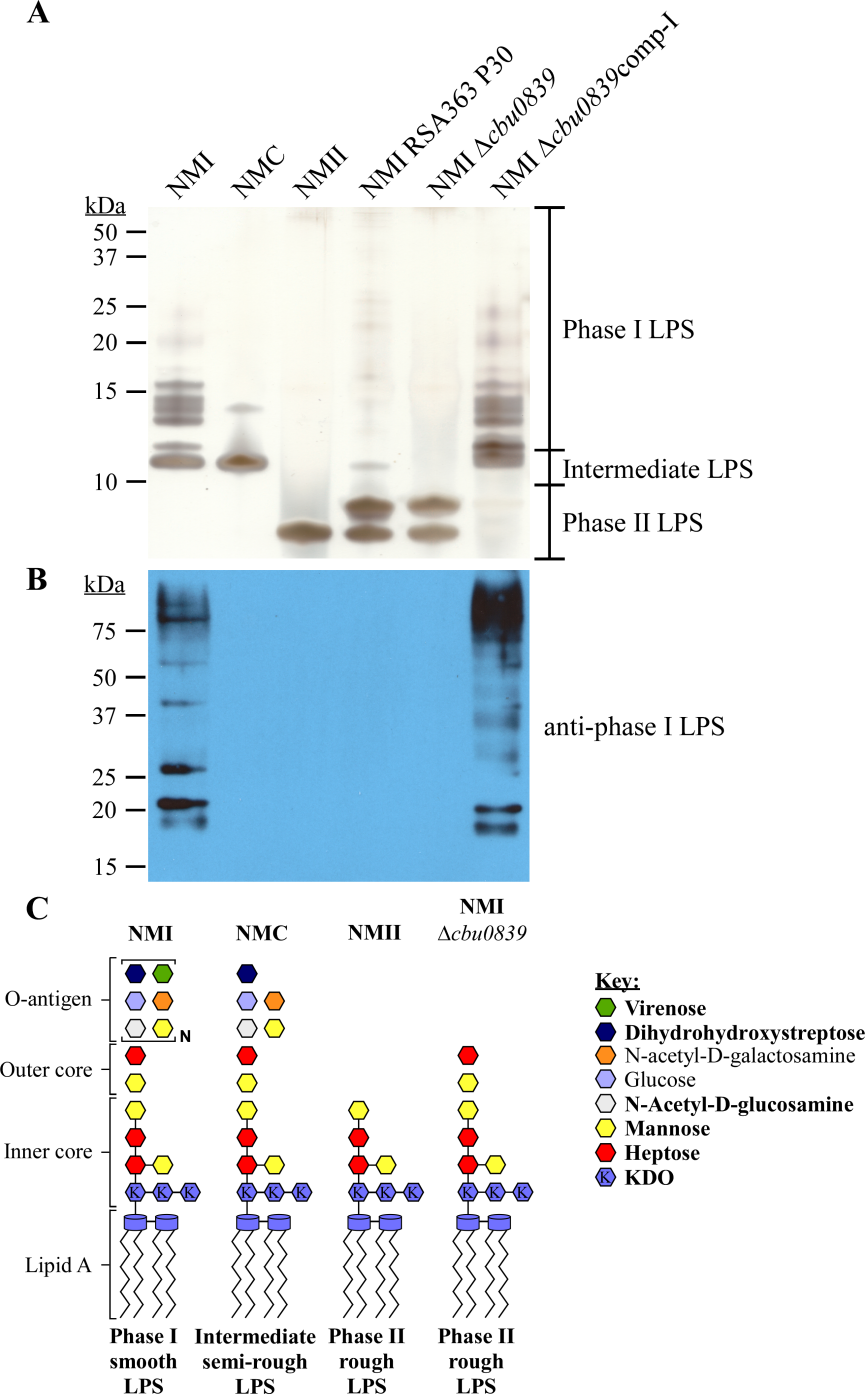
Mutation of *cbu0839* explains the LPS profile of NMI (RSA363) after 30 passages. LPS was extracted from NMI (RSA363) P30, NMI Δ*cbu0839*, and NMI Δ*cbu0839*comp-I following culture in ACCM-D for 7 days. LPS was separated by SDS-PAGE and compared to LPS from NMI, NMC, and NMII by (A) silver stain and (B) immunoblot probed with anti-phase I LPS antibody. Thirty passage NMI (RSA363) contains a mutation in *cbu0839* (Table 1). NMI Δ*cbu0839* and NMI (RSA363) P30 contain phase II LPS by silver stain that does not react with anti-phase I LPS antibody. Expression of a wild type *cbu0839* in NMI Δ*cbu0839* restores production of phase I LPS. These data indicate that mutation of *cbu0839* results in the LPS profile of NMI (RSA363) P30. (C) Model of putative LPS structure of NMI Δ*cbu0839* compared to those of NMI, NMC, and NMII. Bold text in the key indicates highly abundant sugars. KDO is defined as 3-deoxy-D-manno-2-octulosonic acid.

To confirm the *cbu0839* mutation was responsible for phase II LPS, the gene was deleted in NMI (RSA363). The LPS profile of NMI *Δcbu0839* exhibited the ~3 and 6 kDa phase II LPS forms of passage 30 NMI RSA363, with no intermediate or phase I LPS forms (Fig 9A and 9B, S2B Fig). Expression of wild type *cbu0839* in NMI *Δcbu0839* restored production of phase I LPS (Fig 9A and 9B). A model of the predicted LPS structure of NMI Δ*cbu0839* is depicted in Fig 9C. This result indicated that mutations that accumulate in LPS-related genes after in vitro passage are associated with a changing LPS profile. Consistent with this hypothesis, passage of NMC resulted in the accumulation of the same 3 bp deletion in *cbu0533* responsible for phase II LPS of NMII (Table 1, S2A Fig). Interestingly, accumulation of this mutation was also identified in phase II Turkey (RSA315) and Ohio (RSA338), which is a phase II variant of phase I Ohio (RSA270) that lacks this mutation [69, 70], suggesting *cbu0533* is a frequent mutational target in phase variation.

**Fig 10:**
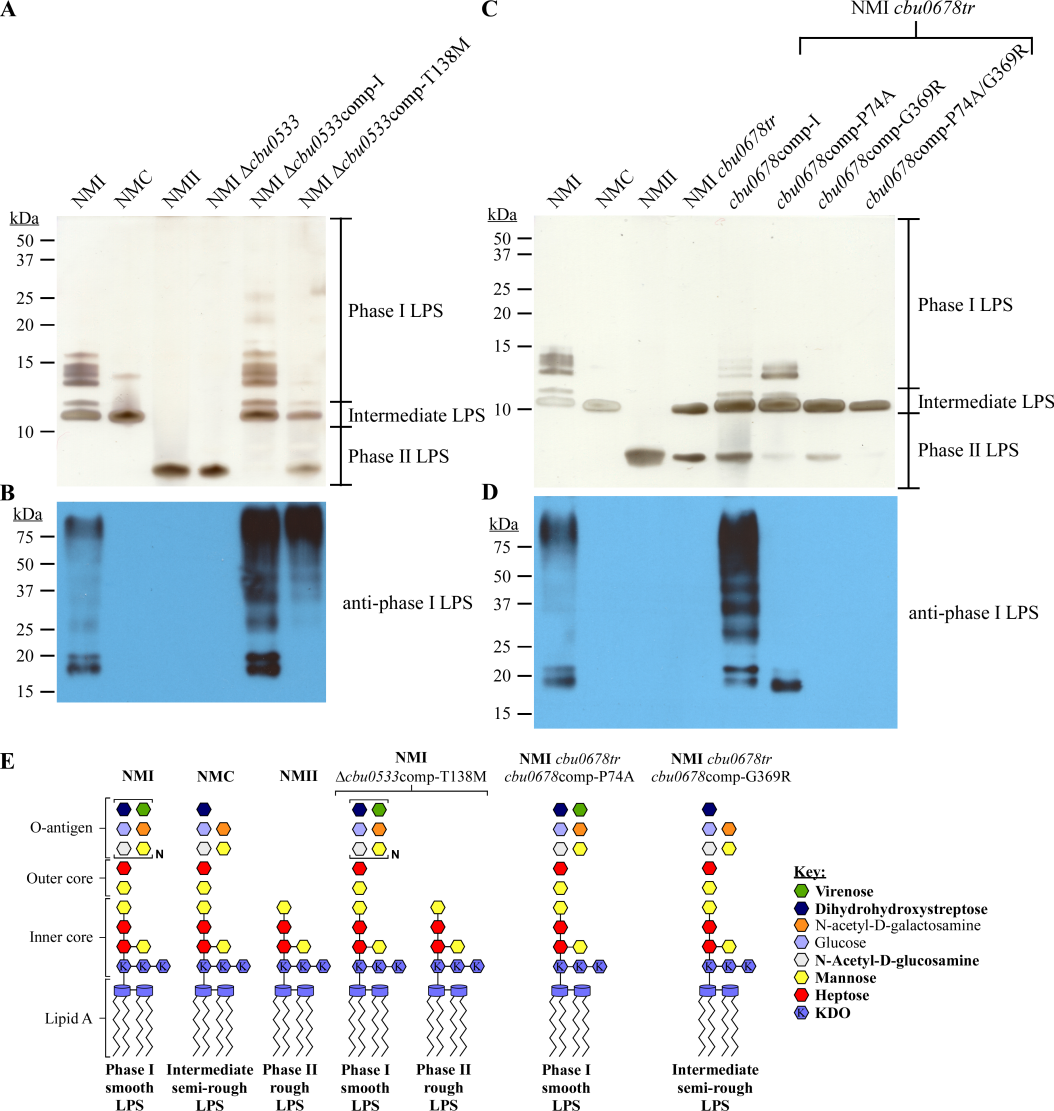
Mutations that accumulate in LPS genes affect protein function. LPS was extracted from NMI Δ*cbu0533*comp-I and NMI Δ*cbu0533*comp-T138M following culture in ACCM-D for 7 days. LPS was separated by SDS-PAGE and compared to LPS from NMI, NMC, and NMII by (A) silver stain or (B) immunoblot probed with anti-phase I LPS antibody. Expression of *cbu0533*-T138M in NMI Δ*cbu0533* fails to fully restore production of phase I LPS, indicating the mutation affects CBU0533 function. LPS was extracted from NMI *cbu0678tr* and NMI *cbu0678tr* expressing wild type *cbu0678*, *cbu0678*comp-P74A, *cbu0678*comp-G369R, or *cbu0678*comp-P74A/G369R following culture in ACCM-2 for 7 days. LPS was separated by SDS-PAGE and compared to LPS from NMI, NMC, and NMII by (C) silver stain or (D) immunoblot probed with anti-phase I LPS antibody. NMI *cbu0678tr* expressing *cbu0678*-P74A produces a modified phase I LPS. NMI *cbu0678tr* expressing *cbu0678*-G369R or *cbu0678*comp-P74A/G369R also does not produce phase I LPS. These data confirm that identified point mutations in *cbu0678* can affect CBU0678 function. (E) Model of putative LPS structures of NMI Δ*cbu0533*comp-T138M, NMI *cbu0678tr cbu0678*comp-P74A, and NMI *cbu0678tr cbu0678*comp-G369R compared to those of NMI, NMC, and NMII. Bold text in the key indicates highly abundant sugars. KDO is defined as 3-deoxy-D-manno-2-octulosonic acid.

Insertion and deletion mutations generally disrupt protein function whereas point mutations can have disparate effects. Sequencing of passaged strains identified point mutations in homologs of *cbu0533* and *cbu0678* in S (Q217) and G (Q212), respectively. The *cbu0533* homolog of S (Q217) accumulated a point mutation conferring a T138M change. The mutational frequency was highest at passage 10 (44.0%), then decreased in passage 20 (16.9%) and 30 (0.6%), which corresponded to the level of phase II LPS (Fig 2 and S2B Fig). To determine the effect of this mutation, *cbu0533*-T138M was expressed in NMI Δ*cbu0533*. Expression of *cbu0533*-T138M did not result in the same phase I LPS profile as expression of wild type *cbu0533* (Fig 10A and 10B). Specifically, LPS bands between 10 and 20 kDa were missing. These data suggested that CBU0533-T138M may retain partial function. The appearance of mutations conferring P74A and G369R changes in domain II and domain I, respectively, of the *cbu0678* homolog of G (Q212), coincided with a phase I to intermediate LPS change (Fig 4A). In *E*. *coli*, these domains function independently of each other, suggesting either mutation could affect enzyme activity [64]. Expression of *cbu0678*-P74A in NMI *cbu0678tr* resulted in LPS lacking most of its O-antigen, with only a single band detected by anti-phase I LPS antibody (Fig 10C and 10D). Expression of *cbu0678*-G369R or the double mutant *cbu0678*-P74A/G369R in NMI *cbu0678tr* failed to restore phase I LPS production (Fig 10C and 10D). Thus, both point mutations affect function of CBU0678. A model of the predicted LPS structures resulting from these mutations is depicted in Fig 10E. Collectively, these results demonstrate that serial passage of *C*. *burnetii* in axenic media causes accumulation of mutations that disrupt LPS biosynthesis genes.

## Discussion

As a critical virulence factor, understanding synthesis and modification of *C*. *burnetii* LPS is essential for expanding our knowledge of Q fever pathogenesis and protective immunity. Here, we identified natural mutational pathways that generate three distinct phase II LPS molecules, two of which are antigenically unique based on antibody reactivity. A fourth deep-rough phase II was generated by gene knockout. Phase II strains grow in eukaryotic host cells, indicating extended LPS structure is unnecessary for lysosomal resistance (S1 Table) [58]. A gene necessary for virenose synthesis and O-antigen addition to an intermediate LPS form was also identified. Organisms synthesizing this form can be derived from a chronically-infected animal (i. e., NMC), as well as during in vitro passage [38]. It is interesting to speculate on whether organisms producing intermediate LPS are found in persistent focalized infections of humans, potentially as a result of defective immune clearance.

This study provides important insight into *C*. *burnetii* phase transition in addition to identifying several steps in LPS biosynthesis. Disparate mutations in *cbu0678* of five *C*. *burnetii* phase II strains indicate this gene is a common target during phase variation. Disruption of *cbu0678* causes full-length LPS of phase I bacteria to convert to an intermediate length LPS similar to that of NMC. The function of CBU0678 is unknown. The enzyme is annotated as a bifunctional sugar kinase/adenylyltransferase with homology to CBU1655. The domain structure of CBU1655 is reversed from CBU0678, and we show it is involved in synthesis of the inner core.

Mutation of *cbu0533* or *cbu0845* causes phase I to phase II LPS transition. Indeed, a 3 base pair deletion in *cbu0533* is solely responsible for the truncated phase II LPS of NMII. This result solves the puzzle as to why NMC, with a 31.5 kb chromosomal deletion of LPS-encoding genes that overlaps the deletion of NMII, produces a larger molecular weight intermediate LPS [46, 49, 52]. The function of CBU0533 is unknown. CBU0533 has homology to WecA, which in *E*. *coli* encodes a undecaprenyl-phosphate alpha-N-acetylglucosamine phosphotransferase that catalyzes the first step in O-antigen synthesis [61]. However, *cbu0533* does not complement an *E*. *coli* Δ*wecA* mutant [31], suggesting it has a different function in *C*. *burnetii*. Consistent with this hypothesis, deletion of *cbu0533* in *C*. *burnetii* results in loss of LPS outer core and O-antigen, suggesting CBU0533 catalyzes the first step in outer core biosynthesis. High frequencies of the same 3 bp deletion were also found in *cbu0533* homologues of phase II Turkey (RSA315) and Ohio (RSA338) [69, 70]. Thus, *cbu0533* is a common mutational target during transition to phase II. Two separate mutations in *cbu0845* were found in two strains of *C*. *burnetii*: California (RSA350) and M44 (RSA461) C1. CBU0845 encodes a protein annotated as a GDP-mannose dehydrogenase. The presence of mannose in the outer core of *C*. *burnetii* LPS [41], and the LPS profile produced by natural *cbu0845* mutants, suggests that CBU0845 is required for mannose production. Collectively, *cbu0533* and *cbu0845* are mutational hotspots for generating phase II LPS.

Insight into additional genetic lesions underlying *C*. *burnetii* phase variation was achieved by weekly serial passage of five strains in axenic media for 30 weeks. LPS profiles show that phase variation in *C*. *burnetii* strains from distinct genomic groups occurs in a similar fashion. Phase II LPS was detected in less than 10 passages in axenic medium, indicating a rapid transition from phase I to phase II. This is consistent with previous work showing phase II LPS after 10 passages of phase I *C*. *burnetii* in embryonated chicken eggs and tissue culture [27, 29]. In the later report, loss of phase I LPS may have been accelerated due to enhanced infectivity of phase II bacteria for cultured host cells, a behavior attributed to the anionic surface charge of phase II bacteria due to the loss of carbohydrate O-antigen [18, 54]. Sequencing of passage variants identified 14 mutations in 11 predicted LPS-associated genes that accumulate in frequency during passage. Novel mutations in *cbu0678* and *cbu0533* were identified in G (Q212) and S (Q217), respectively. Subsequent functional analysis of the gene products indicate that these mutations affect protein function. In many gram-negative bacteria, genes required for core and O-antigen synthesis are linked [71]. *C*. *burnetii* has three chromosomal regions enriched in genes required for O-antigen synthesis. The genes *cbu0825* to *cbu0856*, *cbu0664* to *cbu0704*, and *cbu1831* to *cbu1838* are implicated in synthesis of dihydrohydroxystreptose, virenose, and sugar components of O-antigen, respectively [72]. Consistent with this organization, additional high frequency mutations were found in *cbu0676* and *cbu0677* that are in an operon with *cbu0678* [73]. Based on co-expression with *cbu0678*, we predict mutation of *cbu0676* and *cbu0677* will also result in an intermediate length LPS due to the lack of virenose synthesis. Interestingly, accumulation of mutations in genes not involved in LPS synthesis was less evident, which again suggests a selective advantage during axenic growth for disruption of LPS biosynthesis.

**Fig 11:**
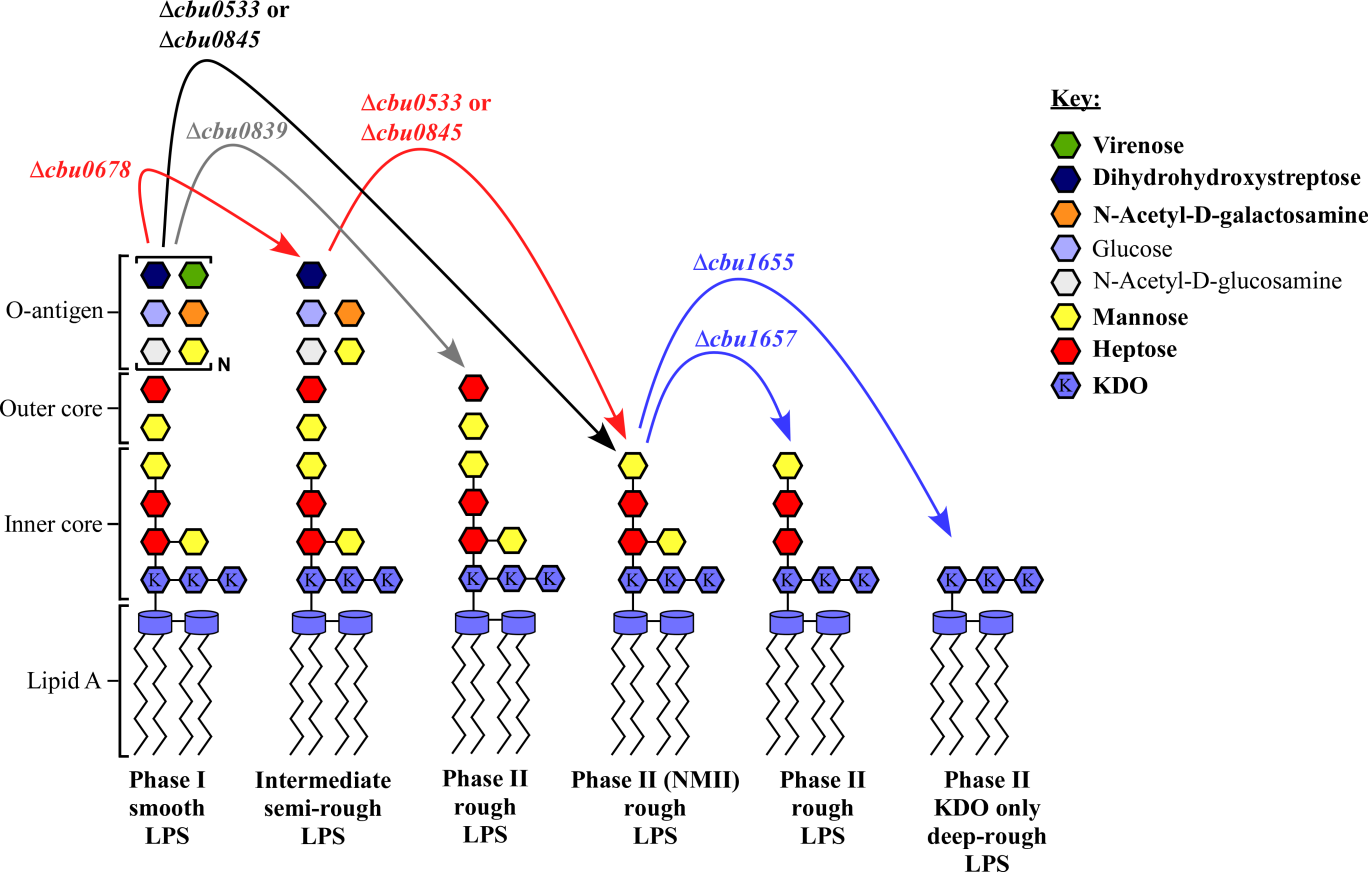
Model of LPS phase transition by *C*. *burnetii*. Depicted are mutational steps occurring during phase variation. Changes in phase transition are shown using putative LPS structures associated with mutation of specific genes. A black arrow indicates mutations that cause direct transition from phase I to phase II LPS, as exemplified by NMII. Red arrows depict mutations that result in transition from phase I to phase II via an intermediate LPS stage, as exemplified by M44 (RSA461) C1. Blue arrows depict additional mutations that modify phase II LPS, as exemplified by Australia (RSA297). The grey arrow indicates transition from phase I to an alternative phase II LPS, as exemplified by NMI (RSA363) following 30 passages in axenic medium. Bold text in the key indicates highly abundant sugars. KDO is defined as 3-deoxy-D-manno-2-octulosonic acid.

With the mutational data identified here, we developed a model for genetic lesions underlying *C*. *burnetii* phase variation (Fig 11). Full-length phase I LPS is required for growth in mammals and potentially arthropods, but not in immunoincompetent host cells or axenic media [18, 30, 74]. Thus, serial passage in axenic media results in the loss of repeating O-antigen. This can occur via accumulation of mutations in genes that result in a semi-rough, intermediate LPS (e.g. *cbu0678*), or alternatively, mutations that directly result in a rough, phase II LPS (e.g. *cbu0839*, *cbu0533*, or *cbu0845*). Transition from a semi-rough intermediate length LPS (e.g. NMC) to a phase II LPS occurs via mutation of *cbu0533* or *cbu0845*. Finally, additional mutations in *cbu1657* and/or *cbu1655* can occur that result in further modification or shortening of phase II LPS structure.

Antibiotic treatment of persistent focal infections can be problematic, and doxycycline-resistant strains have been described [3, 4, 75]. Thus, there is need for new Q fever therapeutics. Increasing antibiotic resistance has resulted in development of small molecule inhibitors that target bacterial LPS and cell wall synthesis [76–80]. The most common LPS targets are LpxA, LpxD, and LpxC, which catalyze the first three steps in lipid A biosynthesis [81]. Additional targets are GmhB and WecA, that are involved in inner core and O-antigen biosynthesis, respectively [82–84]. In *Mycobacterium tuberculosis*, inhibition of WecA by the caprazamycin derivative CPZEN-45 inhibits cell wall biosynthesis [78]. The two *C*. *burnetii* LPS enzymes, CBU0533 and CBU0845, identified in this study as necessary for elongatation of phase II LPS, are potential targets for inhibition. The encoding genes are conserved in all *C*. *burnetii* strains and inhibition of these enzymes in vivo would likely convert phase I to phase II bacteria, which are effectively cleared by the host immune system [18]. Such a treatment might resolve persistent focalized infection with or without antibiotic therapy.

In summary, this study utilized a newly described axenic medium (ACCM-D) [67] and a nutritional selection system to generate directed mutations in *C*. *burnetii* LPS genes. The process for creating gene deletions in virulent *C*. *burnetii* is like that of the avirulent risk group 2 strain NMII [85–87]. A caveat is that the passaging required to expand and clone transformants of phase I *C*. *burnetii* results in a mixed population of organisms producing intermediate or phase II LPS. This problem can be subverted at the cloning stage by picking colonies distinctive of phase I *C*. *burnetii*. Multiple mutational pathways directing phase variation by the organism were revealed. Identification of genes involved in phase II transition allows generation of phase II variants beyond the NMII reference strain. Regulatory approval of these strains for risk group 2 containment would enable host-pathogen studies of *C*. *burnetii* within different genetic groups.

## Methods

### Bacterial strains and mammalian cell lines

The bacterial strains used in this study are listed in S1 Table. Wild type *C*. *burnetii* and genetic transformants were grown microaerobically in ACCM-2 as previously described [88]. For nutritional selection of transformants, strains were grown in ACCM-D minus lysine [67]. *E*. *coli* Stellar (BD Clontech) and PIR1 (ThermoFisher Scientific) cells were used for recombinant DNA procedures and cultivated in Luria broth. *E*. *coli* transformants were selected on LB agar plates containing 50 μg of kanamycin/ml or 10 μg of chloramphenicol/ml. Genes conferring resistance to chloramphenicol, kanamycin, or ampicillin are approved for *C*. *burnetii* genetic transformation studies by the Rocky Mountain Laboratories Institutional Biosafety Committee and the Centers for Disease Control and Prevention, Division of Select Agents and Toxins Program.

### Whole genome sequencing of *C*. *burnetii* strains

Australia (RSA425), Australia (RSA297), California 16 (RSA350), California 16 (RSA350) C2, and M44 (RSA461) C1 were grown in ACCM-2 for 7 days. Nine Mile (RSA363), G (Q212), S (Q217), Dugway (7E65-68), and Nine Mile Crazy (RSA514) were grown in ACCM-2 for 7 days and serially passaged each week for 30 weeks. Genomic DNA was isolated from *C*. *burnetii* strains using the PowerMicrobial Maxi DNA isolation kit (Mo Bio) with an additional step of boiling for 30 minutes prior to physical disruption of the cells. DNA was sequenced using an Illumina MiSeq instrument to generate read pairs as previously described [20, 70].

### Assembly of draft genomes and sequence read archives

Raw FASTQ reads for each sample were quality trimmed using trimmomatic tool, version 0.3 [89] with the following settings: PE ILLUMINCLIP:Truseq3-PE.fa:2:30:10 CROP:225 LEADING:10 TRAILING:10 SLIDINGWINDOW:4:15 MINLEN:36. Quality trimmed reads were then assembled into contiguous sequences (contigs) using SPAdes Genome Assembler, version 3.9.1, and the -careful flag and kmer lengths of 21,33,55,77,99,127. Contigs with coverage less than 2 and shorter than 200 base pairs were discarded. The draft genomes were submitted to Genbank for annotation using the NCBI Prokaryotic Genome Annotation pipeline (PGAP). Annotation stats and accession numbers for each genome are given in S3 Table. Raw sequenced reads for all genomes were submitted to the NCBI sequence read archive (https://www.ncbi.nlm.nih.gov/sra/) with SRA accession numbers for each sample given in S4 Table.

### Identification of mutations in sequenced *C*. *burnetii* genomes

Following trimming, sequence reads were aligned to the Nine Mile RSA493 chromosome (NC_002971.4) and plasmid (NC_004704.2) sequences using Bowtie2 [90]. The SAMtools package was then used to create sorted BAM (Binary Alignment/Map) files [91]. Small nucleotide polymorphisms (SNPs) present in NMI (RSA363), NMII (RSA439), NMC (RSA514), Australia (RSA297), Australia (RSA425), California (RSA350), California (RSA350) C2, and M44 (RSA461) C1 were identified by importing BAM files into Geneious version 10.2.2 (Biomatters). Mutation frequency was determined using the Find Variations/SNPs function with a minimum coverage of 10 and minimum variant frequency of 0.85. BAM files from passaged strains were uploaded into Geneious and analyzed for mutation frequency using the Find Variations/SNPs function with a minimum coverage of 10 and minimum variant frequency of 0.1.

### LPS extraction

*C*. *burnetii* LPS was extracted using a modified hot phenol method as described [52]. LPS from “deep rough” *C*. *burnetii* mutants was isolated using a modified LPS microextraction protocol [65]. Briefly, bacterial cells harvested from a 30 ml ACCM-2 culture were suspended in 100 μL water and boiled for 10 minutes. Samples were cooled, 1 mg of DNaseI (Sigma) added, then incubated for 2 hours at 37°C. One-hundred microliters of lysis buffer [4% SDS, 4% β-mercaptoethanol, 0.1% bromophenol blue, 10% glycerol, 1 M Tris-HCl (pH 6.8)] was added and samples heated at 100°C for 10 minutes. Five microliters proteinase K (20 mg/mL) was added to the cooled samples, which were incubated in a 55°C water bath overnight. Samples were boiled for 5 minutes to inactivate proteinase K.

### Visualization of LPS by silver stain, Gelcode glycoprotein stain, or immunoblotting

Samples for silver or glycoprotein staining were electrophoresed on 16% tricine-SDS-PAGE gels [92]. Samples for immunoblotting were electrophoresed on 12% or 16% glycine-SDS-PAGE gels. LPS bands were sized using the Precision Plus Dual color (Bio-Rad) or SeeBlue Plus2 prestained protein ladders (ThermoFisher Scientific). In gel LPS was stained using SilverQuest or the GelCode Glycoprotein staining kit following the manufacturer’s instructions (ThermoFisher Scientific). For immunoblotting, LPS samples were transferred to PVDF membrane. Mouse monoclonal antibodies AAB-COX-MAB (Bei Resources), 1E4 (a generous gift of Guoguan. Zhang, University of Missouri-Columbia) [93], and A6 [59] were used to label phase I, intermediate, and phase II LPS molecules, respectively. Antibodies were used at dilutions of 1:10000 (AAB-COX-MAB), 1:10000 (1E4), and 1:50 (A6). Reacting LPS was detected using IgG secondary antibody conjugated to horseradish peroxidase and chemiluminescence using Supersignal West Pico chemiluminescent substrate (ThermoFisher Scientific).

### Construction of gene deletion and complementation plasmids

Plasmids and oligonucleotide primers used in this study are listed in S1 and S5 Tables, respectively. Restriction endonucleases were obtained from New England BioLabs. PCR was conducted using Accuprime *Pfx* or *Taq* polymerase (ThermoFisher Scientific). Oligonucleotide primers were purchased from Integrated DNA technologies. All cloning reactions were performed using the In-Fusion HD PCR cloning kit (BD Clontech), and the resulting reactions transformed into either *E*. *coli* PIR1 or Stellar competent cells. Plasmid construction is described in S6 Table. All genetic manipulations of NMII predicted to lengthen LPS and potentially increase virulence were approved by the Rocky Mountain Laboratories Institutional Biosafety Committee and were conducted under risk group 3 conditions.

### Generation and complementation of NMI *cbu0678tr*, NMI Δ*cbu0533*, and NMI Δ*cbu0839*

Nine Mile phase I bacteria were electroporated with 20 μg of suicide plasmid DNA (pJC-Kan::*cbu0678tr*-5'3'-CAT, pJC-CAT::*cbu0533*-5'3'-*lysCA* or pJC-CAT::*cbu0839*-5'3'-*lysCA*) as previously described [88]. Co-integrants were selected by culture of bacteria in ACCM-2 plus 1% FBS containing chloramphenicol (final concentration of 3 μg/ml) and kanamycin (final concentration of 400 μg/ml), or in ACCM-D lacking lysine (ACCM-D-lys) plus 1% FBS. Resolution of pJC-Kan::*cbu0678tr*-5'3'-CAT co-integrants was accomplished by culture for 7 days in ACCM-2 supplemented with chloramphenicol prior to subculture for 4 days in ACCM-2 containing 1% sucrose and chloramphenicol. Resolution of pJC-CAT::*cbu0533*-5'3'-*lysCA* and pJC-CAT::*cbu0839*-5'3'-*lysCA* co-integrants was accomplished by culture in ACCM-D-lys containing 1% sucrose for 4 days. Surviving transformants were expanded by culture in ACCM-2 containing chloramphenicol (NMI *cbu0678tr*) or ACCM-D-lys (NMI Δ*cbu0533* and NMI Δ*cbu0839*) until growth was visible. The NMI *cbu0678tr* mutant was cloned by picking colonies on ACCM-2 agarose as previously described [88]. The NMI Δ*cbu0533* and NMI Δ*cbu0839* mutants were cloned by top spreading 100 μl of diluted 7 day culture on 0.25% ACCM-D-lys agarose followed by a 7 day incubation. Picked bacterial colonies were expanded in their respective media. Verification of NMI *cbu0678tr*, NMI Δ*cbu0533*, and NMI Δ*cbu0839* mutants was conducted by PCR using CBU0678tr-KO-F/CBU0678tr-KO-R, CBU0533-KO-F/CBU0533-KO-R, and CBU0839KO-F/CBU0839KO-R primer pairs, respectively, and gDNA from wild-type or mutant bacteria. Complementation of *C*. *burnetii* NMI *cbu0678tr*, NMI Δ*cbu0533*, and NMI Δ*cbu0839* was achieved by transformation of mutant strains with pJB-Kan::*cbu0678*comp-I, pMiniTn7T-CAT::*cbu0533*comp-I or pMiniTn7T-CAT::*cbu0839*comp-I, respectively [86, 88]. To assess the role of mutations found in NMII *cbu0533*, the mutant S (Q217) homolog of *cbu0533* (T138M), or the active site mutant of NMI *cbu0533* (D156C), NMI Δ*cbu0533* was transformed with pMiniTn7T-CAT::*cbu0533*comp-II, pMiniTn7T-CAT::*cbu0533*comp-T138M, or pMiniTn7T-CAT::*cbu0533*comp-D156C, respectively. Mutations responsible for P74A and G369R changes in the G (Q212) homologue of CBU0678 were examined by transformation of NMI *cbu0678tr* with pJB-Kan::*cbu0678*comp-P74A, pJB-Kan::*cbu0678*comp-G369R or pJB-Kan::*cbu0678*comp-P74A/G369R.

### Generation and complementation of NMII Δ*cbu1655* and NMII Δ*cbu1657*

Generation of *C*. *burnetii* Δ*cbu1655* and Δ*cbu1657* in NMII was achieved using the suicide plasmids pJC-Kan::*cbu1655*-5'3'-CAT and pJC-CAT::*cbu1657*-5'3'-*lysCA* and methods described above for NMI *cbu0678tr* and NMI Δ*cbu0533*, respectively. Verification of NMII Δ*cbu1655* and NMII Δ*cbu1657* was confirmed by PCR of gDNA from wild-type or mutant bacteria using CBU1655-KO-F/CBU1655-KO-R and CBU1657-KO-F/CBU1657-KO-R primer pairs, respectively. *C*. *burnetii* NMII Δ*cbu1655* and NMII Δ*cbu1657* mutants were complemented using pMiniTn7T-Kan::*cbu1655*comp-II and pMiniTn7T-CAT::*cbu1657*comp-II, respectively [86].

### Genetic manipulation of M44 (RSA461) C1, California 16 (RSA350), and Australia (RSA297) strains

M44 (RSA461) C1 was cloned from M44 (RSA459) by plaque assay [60]. California 16 (RSA350) C2 was cloned by micromanipulation [59]. M44 (RSA461) C1, California (RSA350), and California (RSA350) C2 strains were complemented with pMiniTn7T-CAT::*cbu0845*comp-I [86]. Australia (RSA297) was complemented with pJB-CAT::*cbu1657*comp-II [88].

### Accession numbers

The complete genome sequence of *C*. *burnetii* NMC (RSA514), Australia (RSA297), Australia (RSA425), M44 (RSA461) C1, California 16 (RSA350) C2 have been deposited in GenBank under the accession numbers listed in S3 Table. Sequence read archive files of Australia (RSA297), Australia (RSA425), M44 (RSA461) C1, NMI (RSA363), NMC (RSA514), California 16 (RSA350), California 16 (RSA350) C2, Dugway (7E65-68), and passage variants (P2, P10, P20 and P30) of Nine Mile (RSA363), Nine Mile Crazy (RSA514), G (Q212), S (Q217), and Dugway (7E65-68) have been deposited in Genbank under the SRA accession numbers listed in S4 Table.

## Supporting information

S1 Fig NMI and NMII show distinctive growth in axenic media. NMI and NMII were grown for 7 days in (A) liquid ACCM-D or (B) on solid ACCM-D agarose. NMI liquid cultures are considerably less turbid and colonies less defined when compared to NMII. Bar, 100 μm.
(TIF)

S2 Fig Phase transition in strains of different genomic groups following serial passage in axenic media. (A) NMC (RSA514), (B) NMI (RSA363), S (Q217), G (Q212), and Dugway (7E65-68) were passaged weekly in ACCM-2 for 30 weeks. LPS was extracted at passage 2, 10, 20, and 30, separated by SDS-PAGE, then visualized by silver stain or immunoblot probed with LPS-specific antibodies. Passage of NMC results in increasing amounts of phase II LPS. Passage of phase I strains results in decreasing amounts of phase I LPS and increasing amounts of intermediate and phase II LPS.
(TIF)

S3 Fig CBU0533 of NMII has a single amino acid deletion. Alignment of CBU0533 from NMI and NMII compared to *E*. *coli* WecA. The location of CBU0533 amino acid 168 is highlighted in yellow. Active site aspartate residues D156 and D159 of *E*. *coli* WecA are shown in bold.
(TIF)

S4 Fig Gene deletion using a nutritional selection system. (A) *Legionella pneumophilia* and *C*. *burnetii* genes involved in lysine biosynthesis. *C*. *burnetii* is missing the final enzyme (*lysA*) in the pathway. Schematic depicting plasmid integration (B) and excision (C) steps required to replace a targeted gene of interest (GOI) with a lysine cassette (*1169*^*P*^-*lysCA*), which contains the *cbu0678* promoter (*1169*^*P*^) upstream of the fused *lysCA* gene (*lpp0774*) from *L*. *pneumophilia*.
(TIF)

S5 Fig Multiple mutations in CBU0845 are found in phase II strains. Alignment of CBU0845 from NMI compared to homologues in M44 (RSA461) C1 and California 16 (RSA350) C2. Truncations of the protein in California 16 (RSA350) C2 and M44 (RSA461) C1 are shown.
(TIF)

S1 Table Strains and plasmids used in this study. (PDF)

S2 Table Mutations in coding regions of chromosomal genes from *C*. *burnetii* phase II strains identified by whole genome sequencing. (PDF)

S3 Table Features of *C*. *burnetii* draft genomes. (PDF)

S4 Table Accession numbers of sequence read archive submissions of *C*. *burnetii* genomes. (PDF)

S5 Table Oligonucleotides used in this study. (PDF)

S6 Table Construction of plasmids used in this study. (PDF)
