# Supplementary figures and images for: Genetic mechanisms of Coxiella burnetii lipopolysaccharide phase variation
Source: PLoS Pathog. 2018 Feb 26;14(3):e1006922. doi: 10.1371/journal.ppat.1006922 (PMC5843353; doi:10.1371/journal.ppat.1006922)

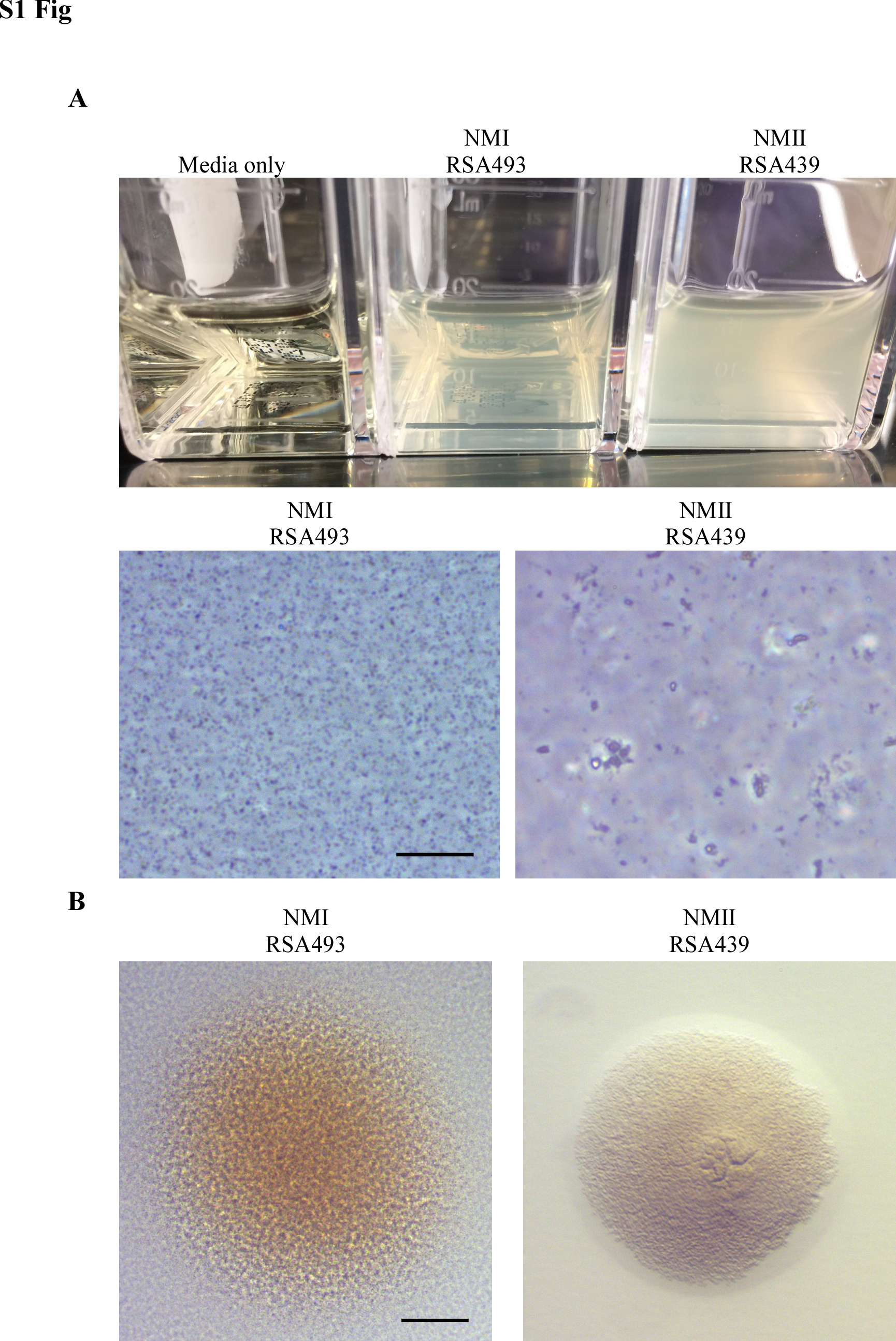

Supplement: S1 Fig — NMI and NMII were grown for 7 days in (A) liquid ACCM-D or (B) on solid ACCM-D agarose. NMI liquid cultures are considerably less turbid and colonies less defined when compared to NMII. Bar, 100 μm. (TIF) [file ppat.1006922.s001.tif]

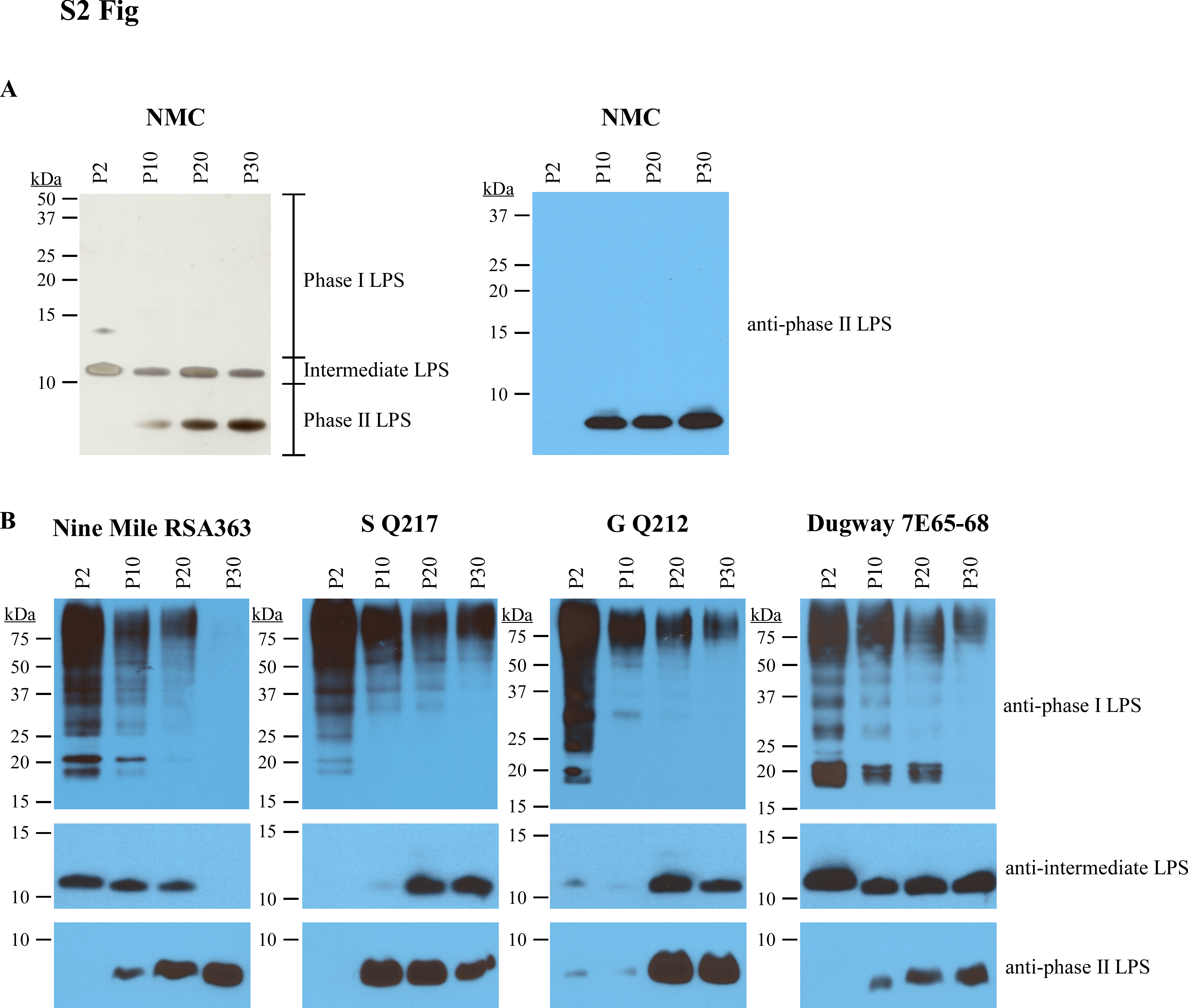

Supplement: S2 Fig — (A) NMC (RSA514), (B) NMI (RSA363), S (Q217), G (Q212), and Dugway (7E65-68) were passaged weekly in ACCM-2 for 30 weeks. LPS was extracted at passage 2, 10, 20, and 30, separated by SDS-PAGE, then visualized by silver stain or immunoblot probed with LPS-specific antibodies. Passage of NMC results in increasing amounts of phase II LPS. Passage of phase I strains results in decreasing amounts of phase I LPS and increasing amounts of intermediate and phase II LPS. (TIF) [file ppat.1006922.s002.tif]

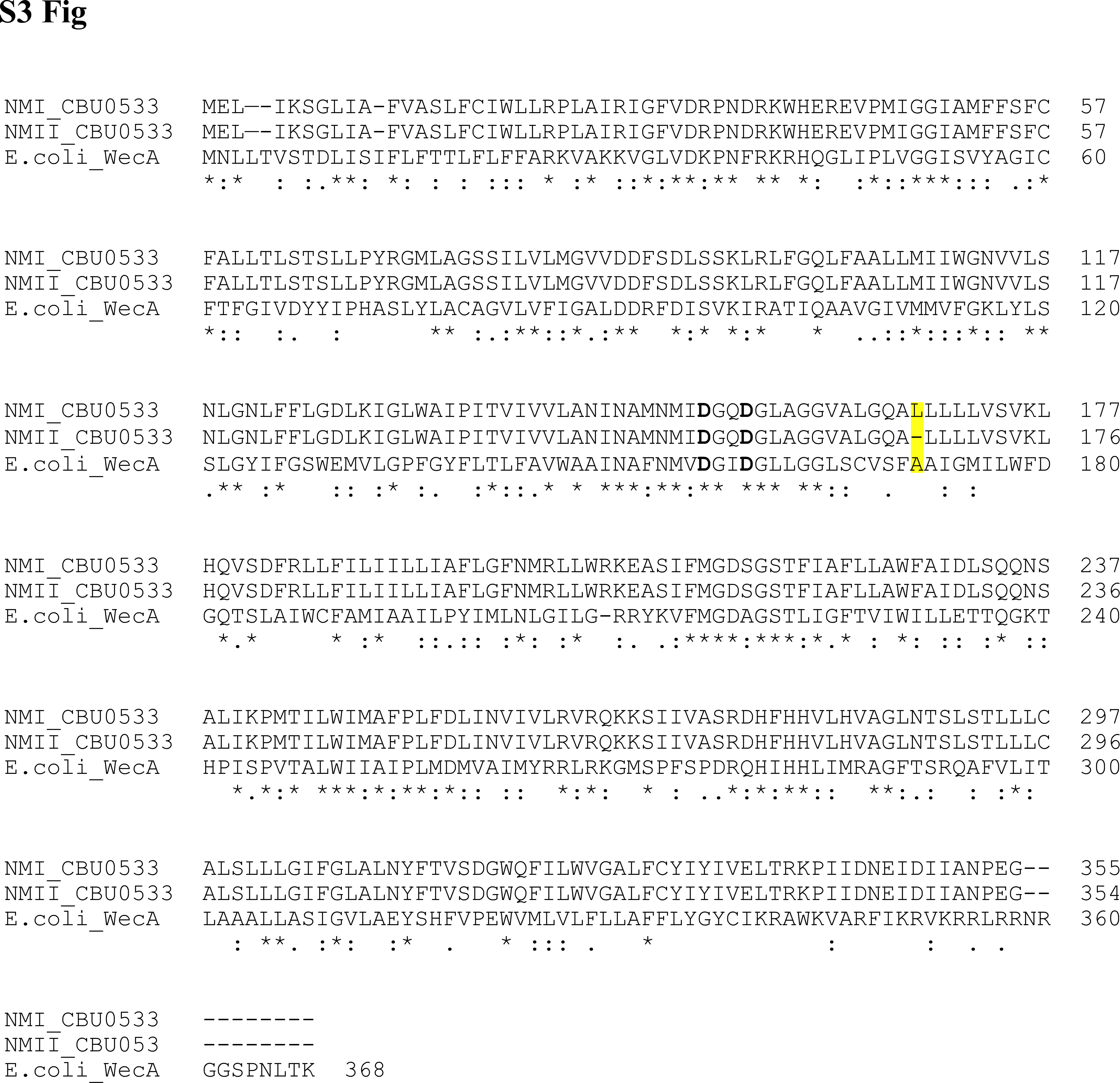

Supplement: S3 Fig — Alignment of CBU0533 from NMI and NMII compared to E. coli WecA. The location of CBU0533 amino acid 168 is highlighted in yellow. Active site aspartate residues D156 and D159 of E. coli WecA are shown in bold. (TIF) [file ppat.1006922.s003.tif]

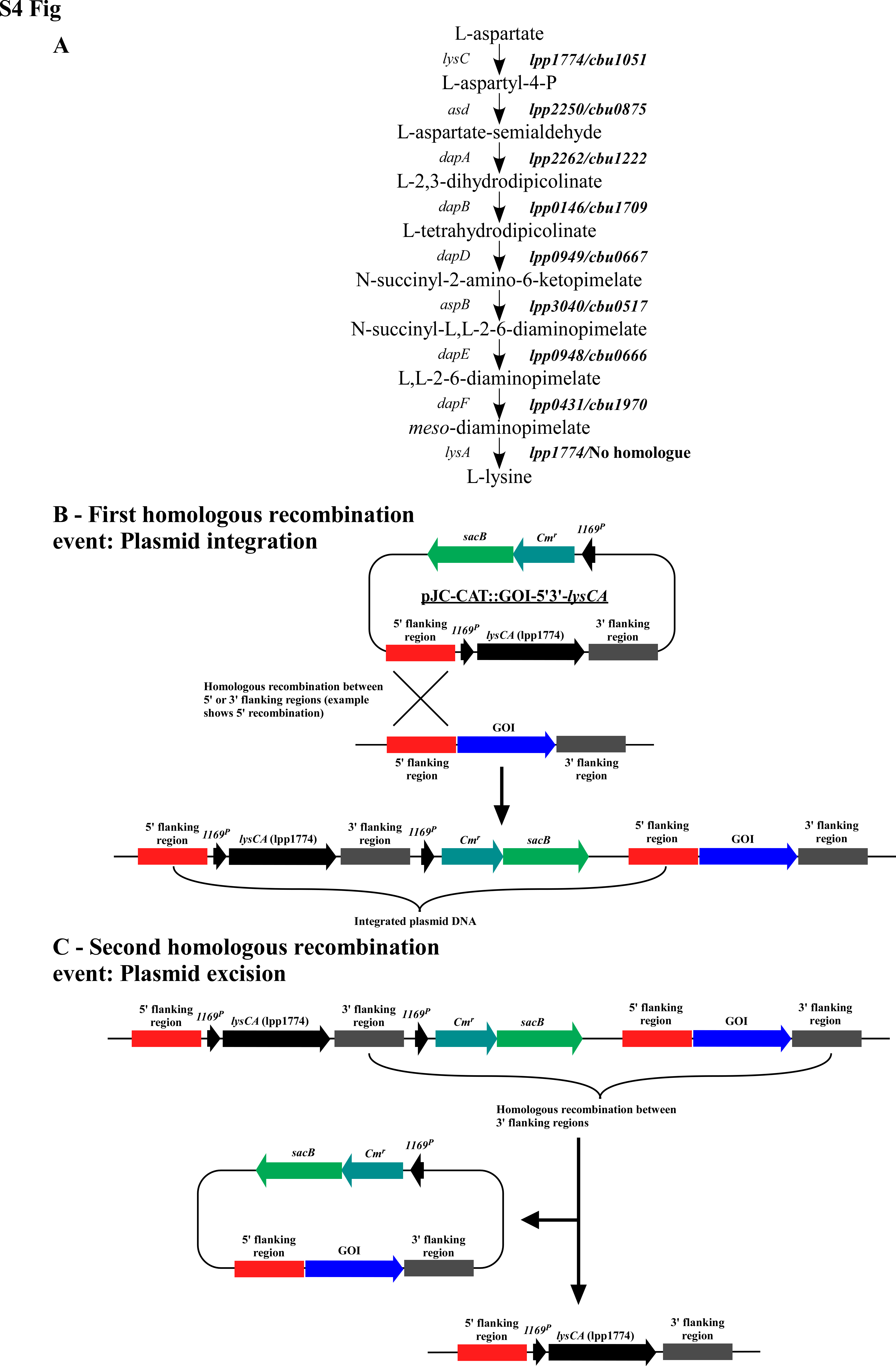

Supplement: S4 Fig — (A) Legionella pneumophilia and C. burnetii genes involved in lysine biosynthesis. C. burnetii is missing the final enzyme (lysA) in the pathway. Schematic depicting plasmid integration (B) and excision (C) steps required to replace a targeted gene of interest (GOI) with a lysine cassette (1169P-lysCA), which contains the cbu0678 promoter (1169P) upstream of the fused lysCA gene (lpp0774) from L. pneumophilia. (TIF) [file ppat.1006922.s004.tif]

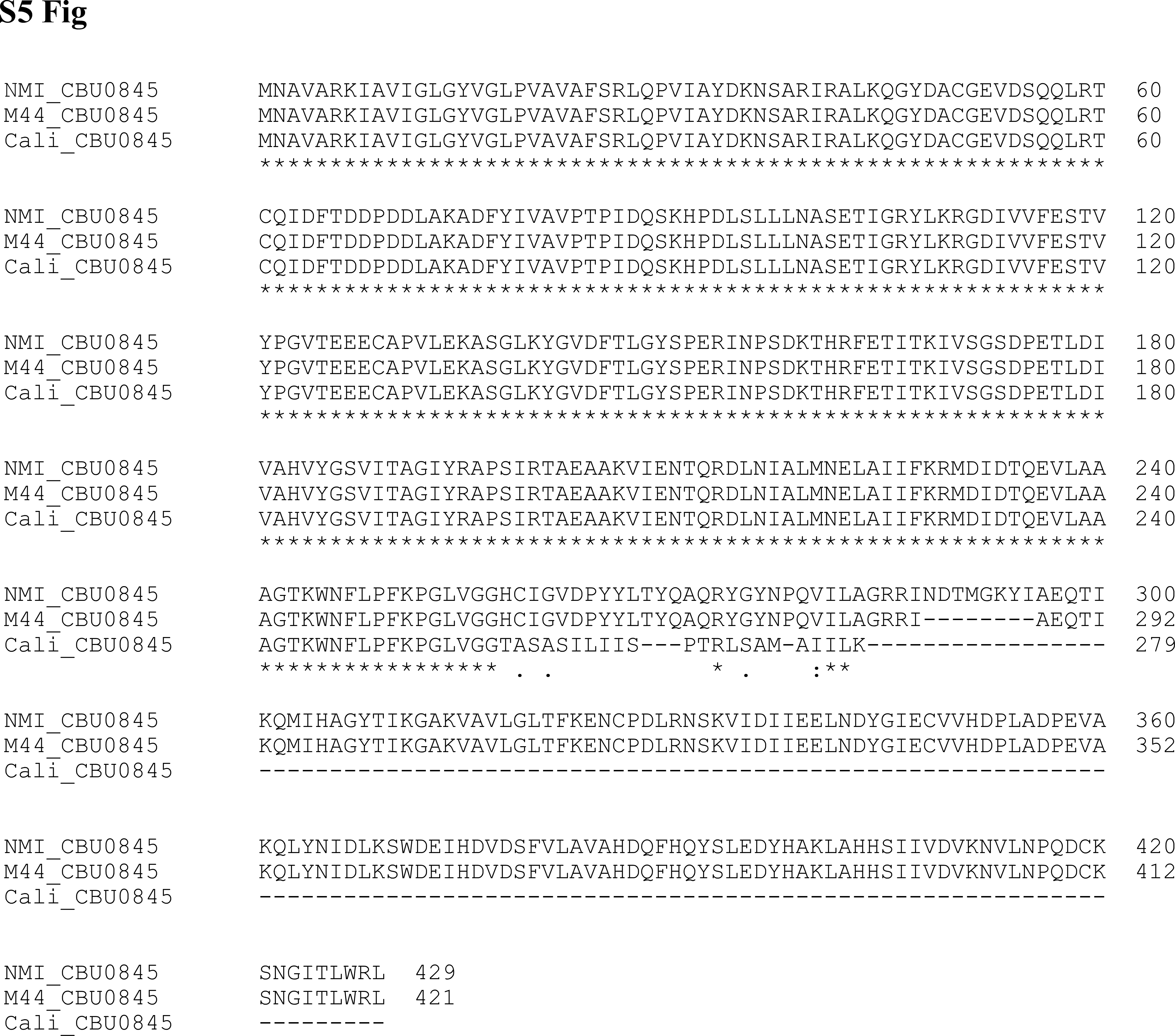

Supplement: S5 Fig — Alignment of CBU0845 from NMI compared to homologues in M44 (RSA461) C1 and California 16 (RSA350) C2. Truncations of the protein in California 16 (RSA350) C2 and M44 (RSA461) C1 are shown. (TIF) [file ppat.1006922.s005.tif]
