# Supplementary material for: Genetic mechanisms of Coxiella burnetii lipopolysaccharide phase variation
Source: PLoS Pathog. 2018 Feb 26;14(3):e1006922. doi: 10.1371/journal.ppat.1006922 (PMC5843353; doi:10.1371/journal.ppat.1006922)
Supplement: S1 Table — (PDF) [file ppat.1006922.s006.pdf]

S1 Table. Bacterial strains and plasmids used in this study

Strain or plasmid	Genotype or strain source	Source/reference
Strains		
E. coli Stellar cells	F–, *endA1*, *supE44*, thi-1, *recA1*, *relA1*, *gyrA96*, *phoA*, Φ80Δ *lacZ*Δ *M15*, Δ (*lacZYA* - *argF*) U169, Δ(*mrr* - *hsdRMS* - *mcrBC*), Δ*mcrA*, λ–	Clontech
E. coli PIR1 cells	F- Δ*lac169, rpoS*(Am), *robA1, creC510, hsdR514, endA, recA1, uidA*(Δ*MluI*)::*pir*-116	Invitrogen
C. burnetii Nine Mile RSA439 (NMII)	Passage variant of Nine Mile RSA493, phase II, clone 4	[1-3]
C. burnetii Nine Mile RSA493 (NMI)	Isolated in Montana (1935) from a tick, phase I, clone 7	[1-3]
C. burnetii Nine Mile RSA363	Passage variant of Nine Mile RSA493, phase I	[1]
C. burnetii Nine Mile Crazy RSA514 (NMC)	Isolated from a chronically infected guinea pig, phase I/II	[4, 5]
C. burnetii Australia (RSA425)	Passage variant of the Australia QD strain RSA425, isolated in Australia (1939) from human blood, phase II	[6]
C. burnetii Australia (RSA297)	Passage variant of the Australia QD strain RSA425, isolated in Australia (1939) from human blood, phase II	[6]
C. burnetii G (Q212)	Isolated in Nova Scotia (1981) from a human heart valve, phase I	[7]
C. burnetii S (Q217)	Isolated in Montana (1981) from a human liver biopsy, phase I	[8]
C. burnetii Dugway (7E65-68)	Isolated in Dugway, Utah (1958) from rodents, phase I	[9, 10]
C. burnetii California 16 (RSA350)	Isolated in California (1947) from cow's milk, phase II	[11, 12]
C. burnetii California 16 (RSA350) C2	Clone 2 of California 16 (RSA350), phase II	This study
C. burnetii M44 C1	Clone 1 of M44 (RSA459), phase II	[2, 13, 14]
C. burnetii cbu0678tr	Nine Mile RSA493, phase I containing a truncated *cbu0678* gene; Cm^r	This study
C. burnetii Δ*cbu1655*	Nine Mile RSA439, phase II containing a *cbu1655* deletion; Cm^r	This study
C. burnetii Δ*cbu0533*	Nine Mile RSA363, phase I containing a *cbu0533* deletion; lysine complemented	This study
C. burnetii Δ*cbu1657*	Nine Mile RSA439, phase II containing a *cbu1657* deletion; lysine complemented	This study
C. burnetii Δ*cbu0839*	Nine Mile RSA363, phase I containing a *cbu0839* deletion; lysine complemented	This study
Plasmids		
pJC-Kan	pJC84 containing *kan* driven by 1169^P; Kan^r	This study
pJC-CAT	pJC84 containing *cat* driven by 1169^P; Cm^r	[15]
pJC-CAT::1169^P-*lysCA*	1169^P-*lysCA* cassette cloned into pJC-CAT; Cm^r	This study
pCR™-Blunt II-TOPO™	Topoisomerase I cloning vector for blunt-ended PCR products, Kan^r	Thermo Fisher Scientific
pCR-BluntII::1169^P-CAT	*loxP-1169P*-CAT-term-*loxP* gBlock fragment cloned into pCR-BluntII-topo	This study
pJC-Kan::*cbu0678tr*-5′3′	5′ and 3′ flanking DNA containing a truncated *cbu0678* cloned into pJC-Kan; Kan^r	This study
pJC-Kan::*cbu0678tr*-5′3′-CAT	1169^P-CAT cassette cloned into pJC-Kan::*cbu0678tr*-5′3′; Cm^r, Kan^r	This study
pJC-Kan::*cbu1655*-5′3′	5′ and 3′ flanking DNA from *cbu1655* cloned into pJC-Kan; Kan^r	This study
pJC-Kan::*cbu1655*-5′3′-CAT	1169^P-CAT cassette cloned into pJC-Kan::*cbu1655*-5′3′; Cm^r, Kan^r	This study
pJC-CAT::*cbu0533*-5′3′	5′ and 3′ flanking DNA from *cbu0533* cloned into pJC-CAT; Cm^r	This study

pJC-CAT::*cbu0533*-5′3′-*lysCA*	*1169*^{P}-*lysCA* cassette cloned into pJC-CAT::*cbu0533*-5′3′; Cm^{r}	This study
pJC-CAT::*cbu1657*-5′3′	5′ and 3′ flanking DNA from *cbu1657* cloned into pJC-CAT; Cm^{r}	This study
pJC-CAT::*cbu1657*-5′3′-*lysCA*	*1169*^{P}-*lysCA* cassette cloned into pJC-CAT::*cbu1657*-5′3′; Cm^{r}	This study
pJC-CAT::*cbu0839*-5′3′	5′ and 3′ flanking DNA from *cbu0839* cloned into pJC-CAT; Cm^{r}	This study
pJC-CAT::*cbu0839*-5′3′-*lysCA*	*1169*^{P}-*lysCA* cassette cloned into pJC-CAT::*cbu0839*-5′3′; Cm^{r}	This study
pJB-Kan-2xHA	pJB2581 containing *kan* driven by *1169*^{P}*;* Kan^{r}	[16]
pJB-CAT	pJB2581 containing *cat* driven by *1169*^{P}*;* Cm^{r}	[16]
pJB-Kan::*cbu0678*comp-I	*Cbu0678* fragment cloned into pJB-Kan-2xHA; Kan^{r}	This study
pJB-Kan::*cbu0678*comp-P74A	*Cbu0678-P74A* fragment cloned into pJB-Kan-2xHA; Kan^{r}	This study
pJB-Kan::*cbu0678*comp-G369R	*Cbu0678-G368R* fragment cloned into pJB-Kan-2xHA; Kan^{r}	This study
pJB-Kan::*cbu0678*comp-P74A/G369R	*Cbu0678-P74A-G369R* fragment cloned into pJB-Kan-2xHA; Kan^{r}	This study
pJB-CAT-2xHA	A 2x hemagglutinin tag cloned into pJB-CAT, Cm^{r}	[17]
pJB-CAT::*cbu1657*comp-II	*Cbu1657*^{P}-*cbu1657* fragment cloned into pJB-CAT-2xHA; Cm^{r}	This study
pTnS2::*1169*^{P}-*tnsABCD*	*cbu1169* promoter cloned into pTnS2; Amp^{r}, R6K *ori*	[18]
pMiniTn7T-CAT	*1169*^{P}-CAT cloned into pUC18R6K-mini-Tn7T-Gm; Cm^{r}, Amp^{r}, R6K *ori*	[18]
pMiniTn7T-Kan	*1169*^{P}-Kan cloned into pUC18R6K-mini-Tn7T-CAT; Kan^{r}, Amp^{r}, R6K *ori*	This study
pMiniTn7T-Kan::*cbu1655*comp-II	*Cbu1655*^{P}-*cbu1655* fragment cloned into pMiniTn7T-Kan; Kan^{r}, Amp^{r}	This study
pMiniTn7T-CAT::*cbu0533*comp-I	*Cbu0533*^{P}-*cbu0533-I*-comp fragment cloned into pMiniTn7T-CAT; Cm^{r}, Amp^{r}	This study
pMiniTn7T-CAT::*cbu0533*comp-II	*Cbu0533*^{P}-*cbu0533-II*-comp fragment cloned into pMiniTn7T-CAT; Cm^{r}, Amp^{r}	This study
pMiniTn7T-CAT::*cbu0533*comp-D156C	*Cbu0533*^{P}-*cbu0533-D156C*-comp fragment cloned into pMiniTn7T-CAT; Cm^{r}, Amp^{r}	This study
pMiniTn7T-CAT::*cbu0533*comp-T138M	*Cbu0533*^{P}-*cbu0533-T138M*-comp fragment cloned into pMiniTn7T-CAT; Cm^{r}, Amp^{r}	This study
pMiniTn7T-CAT::*cbu0845*comp-I	*Cbu0845*^{P}-*cbu0845* fragment cloned into pMiniTn7T-CAT; Cm^{r}, Amp^{r}	This study
pMiniTn7T-CAT::*cbu1657*comp-II	*Cbu1657*^{P}-*cbu1657*comp fragment cloned into pMiniTn7T-CAT; Cm^{r}, Amp^{r}	This study
pMiniTn7T-CAT::*cbu0839*comp-I	*Cbu0839*^{P}-*cbu0839-cbu0840-cbu0841* fragment cloned into pMiniTn7T-CAT; Cm^{r}, Amp^{r}	This study

References

1. Davis GE, Cox HR. A filter-passing infectious agent isolated from ticks. I. Isolation from *Dermacentor andersonii*, reactions with animals, and filtration experiments. Public Health Rep. 1938;53:2259-76.
2. Ormsbee RA, Peacock MG. Rickettsial plaques assay and cloning procedures. Tissue Culture Assoc. 1976;2:475-8.
3. Amano K, Williams JC. Chemical and immunological characterization of lipopolysaccharides from phase I and phase II *Coxiella burnetii*. J Bacteriol. 1984;160(3):994-1002.
4. Peacock MG, Fiset P, Ormsbee RA, Wisseman CL, Jr. Antibody response in man following a small intradermal inoculation with *Coxiella burnetii* phase I vaccine. Acta Virol. 1979;23(1):73-81. PubMed PMID: 35962.

5. Hackstadt T, Peacock MG, Hitchcock PJ, Cole RL. Lipopolysaccharide variation in *Coxiella burnetti*: intrastrain heterogeneity in structure and antigenicity. Infect Immun. 1985;48(2):359-65. PubMed PMID: 3988339; PubMed Central PMCID: PMCPMC261314.
6. Burnet FM, Freeman M. Experimental studies on the virus of "Q" fever. Med J Aust. 1937;2:299-305.
7. Haldane EV, Marrie TJ, Faulkner RS, Lee SH, Cooper JH, MacPherson DD, et al. Endocarditis due to Q fever in Nova Scotia: experience with five patients in 1981-1982. J Infect Dis. 1983;148(6):978-85. PubMed PMID: 6655300.
8. Hendrix LR, Samuel JE, Mallavia LP. Differentiation of *Coxiella burnetii* isolates by analysis of restriction-endonuclease-digested DNA separated by SDS-PAGE. J Gen Microbiol. 1991;137(Pt 2):269-76.
9. Stoenner HG, Lackman DB. The biologic properties of *Coxiella burnetii* isolated from rodents collected in Utah. Am J Hyg. 1960;71:45-51. PubMed PMID: 13834767.
10. Beare PA, Jeffrey BM, Martens CA, Heinzen RA. Draft Genome Sequences of the Avirulent Coxiella burnetii Dugway 7D77-80 and Dugway 7E65-68 Strains Isolated from Rodents in Dugway, Utah. Genome Announc. 2017;5(39). doi: 10.1128/genomeA.00984-17. PubMed PMID: 28963209; PubMed Central PMCID: PMCPMC5624755.
11. Huebner RJ, Jellison WL, Beck MD, Parker RR, Shepard CC. Q fever studies in southern California. I. Recovery of *Rickettsia burneti* from raw milk. Public Health Rep. 1948;63(7):214-22. PubMed PMID: 18899804.
12. Beare PA, Jeffrey BM, Martens CA, Pearson T, Heinzen RA. Draft genome sequences of historical strains of *Coxiella burnetii* isolated from cow's milk and a goat placenta. Genome Announc. 2017;5(39). doi: 10.1128/genomeA.00985-17. PubMed PMID: 28963210; PubMed Central PMCID: PMCPMC5624756.
13. Caminopetros JP. La Q fever en Grèce; le lait source de l'infection pour l'homme et les animaux. Annales de parasitologie humaine et comparee. 1948;23(1-2):107-18. Epub 1948/01/01. PubMed PMID: 18889741.
14. Genig VA. A live vaccine 1-M-44 against Q-fever for oral use. J Hyg Epidemiol Microbiol Immunol. 1968;12(3):265-73. PubMed PMID: 5753316.
15. Beare PA, Larson CL, Gilk SD, Heinzen RA. Two systems for targeted gene deletion in *Coxiella burnetii*. Appl Environ Microbiol. 2012;78(13):4580-9. Epub 2012/04/24. doi: 10.1128/AEM.00881-12. PubMed PMID: 22522687; PubMed Central PMCID: PMC3370473.
16. Omsland A, Beare PA, Hill J, Cockrell DC, Howe D, Hansen B, et al. Isolation from animal tissue and genetic transformation of *Coxiella burnetii* are facilitated by an improved axenic growth medium. Appl Environ Microb. 2011;77(11):3720-5. PubMed PMID: ISI:000290847800021.
17. Beare PA. Genetic manipulation of *Coxiella burnetii*. Advances in experimental medicine and biology. 2012;984:249-71. Epub 2012/06/20. doi: 10.1007/978-94-007-4315-1_13. PubMed PMID: 22711636.
18. Beare PA, Gilk SD, Larson CL, Hill J, Stead CM, Omsland A, et al. Dot/Icm type IVB secretion system requirements for *Coxiella burnetii* growth in human macrophages. Mbio. 2011;2(4):e00175-11. Epub 2011/08/25. doi: 10.1128/mBio.00175-11e00175-11 [pii] mBio.00175-11 [pii]. PubMed PMID: 21862628; PubMed Central PMCID: PMC3163939.
