# Supplementary material for: Genetic mechanisms of Coxiella burnetii lipopolysaccharide phase variation
Source: PLoS Pathog. 2018 Feb 26;14(3):e1006922. doi: 10.1371/journal.ppat.1006922 (PMC5843353; doi:10.1371/journal.ppat.1006922)
Supplement: S2 Table — (PDF) [file ppat.1006922.s007.pdf]

S2 Table. Mutations in coding regions of chromosomal genes from *C. burnetii* phase II strains identified by whole genome sequencing

Locus tag	Function	Gene	Reference location[b]	Reference nucleotide(s)	Test nucleotide(s)	DNA Change[c]	Polymorphism Type[d]	Protein Effect	Codon Change
CBU0032	Hypothetical cytosolic protein		29593	C	T	C -> T	SNP (transition)	None	TCC -> TCT
CBU0032	Hypothetical cytosolic protein		29600	T	G	T -> G	SNP (transversion)	Substitution	TTA -> GTA
CBU0032	Hypothetical cytosolic protein		29634	GGGG	GGGGG	(G)4 -> (G)5	Insertion (tandem repeat)	Frame Shift	
CBU0032	Hypothetical cytosolic protein		29638	T	G	T -> G	SNP (transversion)	None	GGT -> GGG
CBU0032	Hypothetical cytosolic protein		29735	T	C	T -> C	SNP (transition)	None	TTG -> CTG
CBU0032	Hypothetical cytosolic protein		29739	T	C	T -> C	SNP (transition)	Substitution	TTT -> TCT
CBU0032	Hypothetical cytosolic protein		29741	G	A	G -> A	SNP (transition)	Substitution	GTC -> ATC
CBU0049	Hypothetical protein		46839	C	T	C ->T	SNP (transition)	Substitution	GCC -> ACC
CBU0049	Hypothetical protein		47396	T	C	T -> C	SNP (transition)	Substitution	AAA -> AGA
CBU0049	Hypothetical protein		47511	A	G	A -> G	SNP (transition)	Substitution	TCT -> CCT
CBU0062	DnaJ domain protein		56071	G	GCCT	ins (3 bp)	Insertion	Insertion	
CBU0062	DnaJ domain protein		56251	C	G	C -> G	SNP (transversion)	Substitution	CTT -> GTT
CBU0062	DnaJ domain protein		56440	C	T	C -> T	SNP (transition)	Substitution	CTC -> TTC
CBU0062	DnaJ domain protein		57249	GCAT	G	del (3 bp)	Deletion	Inframe deletion	
CBU0136	UDP-N-acetylmuramate--alanine ligase	murC	124181	C	T	C -> T	SNP (transition)	Substitution	GCC -> GTC
CBU0139	Carbonic anhydrase		126357	C	T	C -> T	SNP (transition)	None	CTG -> CTA
CBU0184	Hypothetical membrane associated protein		172737-172803			del (67 bp)	Deletion	Inframe deletion	
CBU0184	Hypothetical membrane associated protein		173015	C	T	C -> T	SNP (transition)	Substitution	TGT -> TAT
CBU0184	Hypothetical membrane associated protein		173284	C	T	C -> T	SNP (transition)	None	CGG -> CGA
CBU0184	Hypothetical membrane associated protein		173613	A	C	A -> C	SNP (transversion)	Substitution	TTT -> GTT
CBU0184	Hypothetical membrane associated protein		173742	TTTTTTT	TTTTTT	(T)7 -> (T)6	Deletion	Inframe deletion	
CBU0303	GTP pyrophosphokinase / Guanosine-3',5'-bis(Diphosphate)	spoT	269704	C	A	C -> A	SNP (transversion)	Substitution	CCC -> CAC
CBU0303	GTP pyrophosphokinase / Guanosine-3',5'-bis(Diphosphate)	spoT	270109	C	T	C -> T	SNP (transition)	Substitution	TCC -> TTC
CBU0311	Outer membrane porin P1		277094	G	A	G -> A	SNP (transition)	Substitution	GGC -> AGC
CBU0311	Outer membrane porin P1		277254	C	T	C -> T	SNP (transition)	Substitution	CCT -> CTT
CBU0311	Outer membrane porin P1		277551	TCTTCAGCTACTG	T	del (12 bp)	Deletion (tandem repeat)	Inframe deletion	
CBU0372	Fic family protein		335984-336350			del (367 bp)	Deletion	Deletion	
CBU0372	Fic family protein		336658	G	A	G -> A	SNP (transition)	Substitution	TCA -> TTA
CBU0390	Virulence factor mviN	mviN	353375	G	A	G -> A	SNP (transition)	Substitution	GCC -> ACC
CBU0533	Undecaprenyl-phosphate alpha-N-acetylglucosaminephosph	rfe	477696	T	C	T -> C	SNP (transition)	Substitution	TCT -> CCT
CBU0533	Undecaprenyl-phosphate alpha-N-acetylglucosaminephosph	rfe	478159	CTAT	C	del (3 bp)	Deletion	Inframe deletion	
CBU0547	Tetratricopeptide repeat family protein		496565	G	T	G -> T	SNP (transversion)	Substitution	GCC -> GAC
CBU0547	Tetratricopeptide repeat family protein		495756	A	G	A -> G	SNP (transition)	None	
CBU0547	Tetratricopeptide repeat family protein		495799	T	C	T -> C	SNP (transition)	None	
CBU0571	Hypothetical exported protein		521492	T	TTTTA	T -> TTTTA	Insertion	Truncation	
CBU0571	Hypothetical exported protein		521647	C	T	C -> T	SNP (transition)	Substitution	GGA -> AGA
CBU0571	Hypothetical exported protein		521694	C	T	C -> T	SNP (transition)	Substitution	AGC -> AAC
CBU0596a	Hypothetical protein		544354	CCCCC	CCCC	(C)5 -> (C)4	Deletion (tandem repeat)	Frame Shift	
CBU0676-CBU0700			617558-649125			del (31,568 bp)	Deletion	Deletion	
CBU0678	D-glycero-D-manno-heptose-1-phosphate adenylyltransferase / D-glyce		620026-620036	CGTACGTCCATGA	C	del (11 bp)	Deletion	Deletion	
CBU0678	D-glycero-D-manno-heptose-1-phosphate adenylyltransferase / D-glyce		620633	T	TA	A -> AA	Insertion	Truncation	
CBU0678	D-glycero-D-manno-heptose-1-phosphate adenylyltransferase / D-glyce		620634	TT	TTT	T -> TT	Insertion (tandem repeat)	Frame Shift	
CBU0678-CBU0698			620667-646663			del (25,997 bp)	Deletion	Deletion	
CBU0686	Pyruvate dehydrogenase E1 component beta subunit		631840	T	C	T -> C	SNP (transition)	None	
CBU0686	Pyruvate dehydrogenase E1 component beta subunit		632107	A	G	A -> G	SNP (transition)	None	TTA -> TTG
CBU0686	Pyruvate dehydrogenase E1 component beta subunit		632110	A	C	A -> C	SNP (transversion)	None	ATA -> ATC
CBU0686	Pyruvate dehydrogenase E1 component beta subunit		632113	C	T	C -> T	SNP (transition)	None	GAC -> GAT
CBU0736	Hypothetical exported protein		678734	T	C	T -> C	SNP (transition)	Substitution	ACA -> GCA
CBU0736	Hypothetical exported protein		678777	G	T	G -> T	SNP (transversion)	Truncation	
CBU0766	Acetoacetyl-CoA synthetase		710186	A	G	A -> G	SNP (transition)	Substitution	TCG -> CCG
CBU0766	Acetoacetyl-CoA synthetase		710512	G	T	G -> T	SNP (transversion)	Substitution	GCA -> GAA
CBU0766	Acetoacetyl-CoA synthetase		710750	C	T	C -> T	SNP (transition)	Substitution	GTA -> ATA
CBU0768	Multidrug resistance protein B		712549	A	G	A -> G	SNP (transition)	Substitution	GAA -> GGA
CBU0768	Multidrug resistance protein B		712654	G	C	G -> C	SNP (transversion)	Substitution	GGG -> GCG

CBU0768	Multidrug resistance protein B		712668	GGG	GG	(G)3 -> (G)2	Deletion (tandem repeat)	Frame Shift	
CBU0768	Multidrug resistance protein B		712955	A	G	A -> G	SNP (transition)	None	
CBU0780	Response regulator GacA	gacA.2	722696	G	A	G -> A	SNP (transition)	Substitution	GCT -> ACT
CBU0780	Response regulator GacA	gacA.2	722718	G	A	G -> A	SNP (transition)	Substitution	CGT -> CAT
CBU0845	UDP-N-acetyl-D-galactosamine 6-dehydrogenase		799572-799595			del (24 bp)	Deletion	Deletion	
CBU0845	UDP-N-acetyl-D-galactosamine 6-dehydrogenase		799682	CCCCCCC	CCCCCC	(C)7 -> (C)6	Deletion (tandem repeat)	Frame Shift	
CBU0845	UDP-N-acetyl-D-galactosamine 6-dehydrogenase		799933	C	G	C -> G	SNP (transversion)	Substitution	GAT -> CAT
CBU0848	Glucose-6-phosphate isomerase / Glucose-6 phosphate 1-ep	pgi	803532	A	G	A -> G	SNP (transition)	Substitution	ATG -> GTG
CBU0918	Hypothetical protein		866895	G	A	G -> A	SNP (transition)	Substitution	GAG -> AAG
CBU0918	Hypothetical protein		867270	G	T	G -> T	SNP (transversion)	Truncation	
CBU0918	Hypothetical protein		867286-867530			del (245 bp)	Deletion	Frame Shift	
CBU0918	Hypothetical protein		867868	A	C	A -> C	SNP (transversion)	Substitution	AAA -> ACA
CBU0918	Hypothetical protein		868118-868411			del (294 bp)	Deletion	Deletion	
CBU1034	CDP-diacylglycerol--glycerol-3-phosphate 3-phosphatidyltra	pgsA	976728	G	A	G -> A	SNP (transition)	Substitution	GCC -> GTC
CBU1034	CDP-diacylglycerol--glycerol-3-phosphate 3-phosphatidyltra	pgsA	976743	C	A	C -> A	SNP (transversion)	Substitution	TGG -> TTG
CBU1043	Response regulator GacA	gacA.4	985013	TAT	T	TAT -> T	Deletion	Frame Shift	
CBU1084	Two component system histidine kinase		1028854	A	C	A -> C	SNP (transversion)	Substitution	GTT -> GGT
CBU1084	Two component system histidine kinase		1029078-1029267			del (190 bp)	Deletion	Deletion	
CBU1084	Two component system histidine kinase		1029452	G	A	G -> A	SNP (transition)	Substitution	CGC -> TGC
CBU1084	Two component system histidine kinase		1029577	T	C	T -> C	SNP (transition)	Substitution	CAC -> CGC
CBU1084	Two component system histidine kinase		1029744	A	G	A -> G	SNP (transition)	None	
CBU1131	RRNA methylase, SpoU family		1074222	T	C	T -> C	SNP (transition)	Substitution	ATA -> ATG
CBU1131	RRNA methylase, SpoU family		1074407	G	C	G -> C	SNP (transversion)	Substitution	CCA -> GCA
CBU1176	Deoxyribodipyrimidine photolyase	phrB	1118388	GT	G	GT -> G	Deletion	Frame Shift	
CBU1176	Deoxyribodipyrimidine photolyase	phrB	1118873	G	A	G -> A	SNP (transition)	None	
CBU1176	Deoxyribodipyrimidine photolyase	phrB	1119417	AAAA	AAA	(A)4 -> (A)3	Deletion (tandem repeat)	Frame Shift	
CBU1176	Deoxyribodipyrimidine photolyase	phrB	1119539	C	T	C -> T	SNP (transition)	None	
CBU1537	Non-proteolytic protein, peptidase family M23		1486809	G	A	G -> A	SNP (transition)	Substitution	GGC -> AGC
CBU1634	IcmQ protein	icmQ	1574754	C	T	C -> T	SNP (transition)	Substitution	AGC -> AAC
CBU1657	alpha-L-glycero-D-manno-heptose beta-1,4-glucosyltransferase		1594819	TTTTTTTTT	TTTTTTTT	(T)9 -> (T)8	Deletion (tandem repeat)	Frame Shift	
CBU1723	Thiol:disulfide interchange protein DsbD	dsbD	1655918-1656340			del (423 bp)	Deletion	Deletion	
CBU1723	Thiol:disulfide interchange protein DsbD	dsbD	1656265	G	A	G -> A	SNP (transition)	Truncation	
CBU1724	Hypothetical protein		1657496	T	C	T -> C	SNP (transition)	Substitution	GAG -> GGG
CBU1724	Hypothetical protein		1657498	C	T	C -> T	SNP (transition)	None	
CBU1724	Hypothetical protein		1657670	AAA	AA	(A)3 -> (A)2	Deletion (tandem repeat)	Frame Shift	
CBU1724	Hypothetical protein		1659294	G	A	G -> A	SNP (transition)	Substitution	CTC -> TTC
CBU1772	GTP-binding protein YihA	yihA	1705067	T	C	T -> C	SNP (transition)	Substitution	AAG -> GAG

Notes

[a] - Nucleotide(s) differences identified in sequence reads of test genomes compared to Nine Mile (RSA493), del = the presence of a deletion, (mix) - indicates a mixed population of wild type and mutant nucleotides.

[b] - Nucleotide location of polymorphism relative to the Nine Mile (RSA493) genome

[c] - del - Presence of deletion in the test genome, deletion size indicated in parentheses.

[d] - SNP - small nucleotide polymorphism

[e] - mutations in common genes of Genomic group I strains are listed for M44 RSA461 C1, unique mutations in other genes are not listed.

	Reference genome	Test genomes nucleotide(s)[a]							
**Amino Acid Change**	**NMI (RSA493)**	**NMI (RSA363)**	**NMII RSA439**	**NMC (RSA514)**	**Australia (RSA297)**	**Australia (RSA425)**	**California 16 (RSA350)**	**California 16 (RSA350) C2**	**M44 (RSA46) C1[e]**
	C						T	T	
L -> V	T						G	G	
	GGGG						GGGGG	GGGGG	GGGGG
	T						G	G	G
	T						C	C	C
F -> S	T						C	C	C
V -> I	G						A	A	A
A -> T	C								T
K -> R	T								C
S -> P	A				G	G			
S -> SP	G								GCCT
L -> V	C								G
L -> F	C								T
	GCAT						G	G	
A -> V	C	T	T	T	T	T			
	C	T	T	T	T	T			
									del
C -> Y	C								T
	C	T	T	T	T	T			
F -> V	A								C
	TTTTTTT								TTTTTT
P -> H	C				A	A			
S -> F	C								T
G -> S	G		A						
P -> L	C								T
	TCTTCAGCTACTG				T	T			
									del
S -> L	G	A	A	A	A	A	A	A	A
A -> T	G	A							
S -> P	T				C	C			
	CTAT		C						
A -> D	G	T	T	T					
	A								G
	T								C
	T								TTTTA
G -> R	C								T
S -> N	C						T	T	
	CCCCC	CCCC	CCCC	CCCC	CCCC	CCCC			
				del					
	CGTACGTCCATGA								C
	A						AA (mix)	AA	
	T				TT	TT			
			del						
	T								C
	A	G				G			
	A	C				C			
	C	T				T			
T -> A	T								C
	G						T	T	
S -> P	A								G
A -> E	G	T	T	T					
V -> I	C								T
E -> G	A								G
G -> A	G	C	C	C	C	C			

	GGG								CC
	A								G
A -> T	G								A
R -> H	G				A	A			
									del
	CCCCCCC						CCCCCC (mix)	CCCCCC	
D -> H	C								G
M -> V	A						G	G	
E -> K	G								A
	G								T
		del	del	del					
K -> T	A								C
									del
A -> V	G						A	A	
W -> L	C								A
	TAT	T	T	T					
V -> G	A						C	C	
					del	del			
R -> C	G	A	A	A					
H -> R	T	C	C	C	C	C	C	C	C
	A								G
I -> M	T								C
P -> A	G						C	C	C
	GT								G
	G								A
	AAAA	AAA	AAA	AAA	AAA	AAA			
	C								T
G -> S	G						A	A	
S -> N	C	T	T	T	T	T			
	TTTTTTTTT				TTTTTTTT	TTTTTTTT (mix)			
									del
	G					A (mix)			
E -> G	T						T	T	C
	C								T
	AAA								AA
L -> F	G								A
K -> E	T	C	C	C	C	C	C	C	C
