# Supplementary material for: Genetic mechanisms of Coxiella burnetii lipopolysaccharide phase variation
Source: PLoS Pathog. 2018 Feb 26;14(3):e1006922. doi: 10.1371/journal.ppat.1006922 (PMC5843353; doi:10.1371/journal.ppat.1006922)
Supplement: S3 Table — (PDF) [file ppat.1006922.s008.pdf]

S3 Table. Features of *C. burentii* draft genomes

Strain	Genome accession no.	No. of contigs	Genome coverage (x)	Plasmid coverage (x)	Chromosome size (bp)	Total no. of chromosome genes *	Plasmid size (bp)	Total no. of plasmid genes
Australia RSA297	NOVK00000000	34	75	104	1,969,029	2,143	37,446	50
Australia RSA425	NOVJ00000000	34	133	160	1,969,016	2,144	37,219	50
M44 RSA461 C1	NOVI00000000	66	80	103	1,968,956	2,168	37,451	50
Nine Mile RSA363	NOVH00000000	35	113	150	1,969,086	2,144	37,446	50
Nine Mile Crazy RSA514	NOVG00000000	33	433	157	1,935,931	2,136	37,374	50
California 16 RSA350 clone 2	NOVF00000000	34	125	172	1968794	2,145	37,446	50

* Total gene count includes coding genes, RNAs (tRNAs, noncoding RNAs [ncRNAs], and rRNAs), and pseudogenes.
