# Supplementary material for: Genetic mechanisms of Coxiella burnetii lipopolysaccharide phase variation
Source: PLoS Pathog. 2018 Feb 26;14(3):e1006922. doi: 10.1371/journal.ppat.1006922 (PMC5843353; doi:10.1371/journal.ppat.1006922)
Supplement: S4 Table — (PDF) [file ppat.1006922.s009.pdf]

S4 Table. Accession numbers of sequence read archive submissions of *C. burnetii* genomes

	SRA accession no.
Australia RSA297	SRR6300873
Australia RSA425	SRR6300869
M44 RSA461 Clone 1	SRR6300871
Nine Mile RSA363	SRR6300872
Nine Mile Crazy RSA514	SRR6300868
California 16 RSA350 clone 2	SRR6300870
California 16 RSA350	SRR6300874
Dugway 7E65-68	SRR6300875
Nine Mile RSA363 passage 2	SRR6300860
Nine Mile RSA363 passage 10	SRR6300861
Nine Mile RSA363 passage 20	SRR6300866
Nine Mile RSA363 passage 30	SRR6300867
Nine Mile Crazy RSA514 passage 2	SRR6300864
Nine Mile Crazy RSA514 passage 10	SRR6300865
Nine Mile Crazy RSA514 passage 20	SRR6300858
Nine Mile Crazy RSA514 passage 30	SRR6300859
Dugway 7E65-68 passage 2	SRR6300876
Dugway 7E65-68 passage 10	SRR6300877
Dugway 7E65-68 passage 20	SRR6300862
Dugway 7E65-68 passage 30	SRR6300863
CbuG_Q212 passage 2	SRR6300879
CbuG_Q212 passage 10	SRR6300878
CbuG_Q212 passage 20	SRR6300881
CbuG_Q212 passage 30	SRR6300880
CbuS_Q217 passage 2	SRR6300883
CbuS_Q217 passage 10	SRR6300882
CbuS_Q217 passage 20	SRR6300885
CbuS_Q217 passage 30	SRR6300884
