# Supplementary material for: Genetic mechanisms of Coxiella burnetii lipopolysaccharide phase variation
Source: PLoS Pathog. 2018 Feb 26;14(3):e1006922. doi: 10.1371/journal.ppat.1006922 (PMC5843353; doi:10.1371/journal.ppat.1006922)
Supplement: S5 Table — (PDF) [file ppat.1006922.s010.pdf]

S5 Table. Oligonucleotide primers used in this study

Primer	Sequence (5′ to 3′)
Primers for pJC-Kan construction	
Kan-forpJC-F	TGAAGGAGGGCCCTTATGATTGAACAAGATGGATTGCACGC
Kan-forpJC-R	CATGTCTCCTGCTAGCTTATCAGAAGAACTCGTCAAGAAGGC
Primers for pMiniTn7T-Kan construction	
1169P-Kan-forTn7T-F	TATCGATACCGTCGACATGGCTTCGTTTCGCAGCGAAC
1169P-Kan-forTn7T-R	GGGGTTCGAGGTCGACTTATCAGAAGAACTCGTCAAGAAGG
Primers for *1169P-lysCA* cassette construction	
LysCA-F	CTTCATGAAGGAGGCTGCAGTTGAGCGGAATACTAATCATGCAAC
LysCA-R	GAACCTGTTTGTCGACATAACTTCGTATAATGTATGCTATACGAAGTTATTTATTCCAG AACTATTTCCTGAG
1169P-forLysCA-F	CGGTACCCGGGGATCCATAACTTCGTATAGCATACATTATACGAAGTTATATGGCTTC GTTTCGCAGCGAACTTG
1169P-forLysCA-R	CCTCCTTCATGAAGG
Primers for gene deletion, analysis, and complementation	
CBU0678tr-5′-F	CGGTACCCGGGGATCCTGTTTGAAAATGTTGATTATGTTTTCC
CBU0678tr-5′-R	CACCCATATGCGACGCGAGCGTCGAGAATGAATCGAGGGCTTCATGATCGC
CBU0678tr-3′-F	CGTCGCATATGGGTGCGCATGTACGTCCGATAAAAGAAAATGAGACGCTGGTAAC
CBU0678tr-3′-R	GAACCTGTTTGTCGACCACAGCAGGGTCATGCACCTGTATC
CBU0678tr-KO-F	GGAATTATGTGAAATAGTGGGTCAATTC
CBU0678tr-KO-R	GGATCGTGTTGTTGACTGTGTG
CBU0678tr-comp-F	CTTCATGAAGGAGGCTGCAGATGTTGCTGAAACGATACCGTC
CBU0678tr-comp-R	TCGTATGGGTACATCTGCAGATAGCGAATGACTTCATATTTAGG
CBU0678-P74A-comp-F	GCTCACATTCCGCAAGATTTGCG
CBU0678-P74A-comp-R	TTGCGGAATGTGAGCACGATAATGCCCCTTAACAATG
CBU0678-G369R-comp-F	AGAAATATAACCGAATTTAGAAATTTCG
CBU0678-G369R-comp-R	TTCGGTTATATTTCTCCACCGACTTGCCACCTGAC
1169P-CAT-NdeI-F	GCTCGCGTCGCATATGATAACTTCGTATAGCATACATTATACGAAGTTATATGGCTTC GTTTCGCAGC
1169P-CAT-NdeI-R	CATGCGCACCCATATGATAACTTCGTATAATGTATGCTATACGAAGTTATTATAAACG CAGAAAGGCCCAC
CBU1655-5′-F	CGGTACCCGGGGATCCCATTGATTTAATTTACCTCGATTGGC
CBU1655-5′-R	CACCCATATGCGACGCGAGCGTCGAGTCAAAGACTAAAGAGGCAGC
CBU1655-3′-F	CGTCGCATATGGGTGCGCATGTACGTCGCGGTTATCTTAATTTAATCTACTC
CBU1655-3′-R	GAACCTGTTTGTCGACAACATCAGCGTTGGCGGACATTG
CBU1655-KO-F	GTGGCATAATAAGCGCTCGTTTC
CBU1655-KO-R	CGATTTGGCTGGGTTGGTAATCGC
CBU1655-comp-F	GCTTCTCGAGGAATTCTTCGCTCCTACCAGGCAATC
CBU1655-comp-R	TTACTCAATGGAATTCAACCAAAGCAGTGCTTGTAACC
CBU0533-5′-F	CGGTACCCGGGGATCCCAGACCCAAAAGTTATTGTGGC
CBU0533-5′-R	CACCCATATGCGACGCGAGCGTCGAGAAAAAGTCACATCCTGCAGTTCT
CBU0533-3′-F	CGTCGCATATGGGTGCGCATGTACGTCGCTCTTGTGACTAAAACCTCC

CBU0533-3′-R	GAACCTGTTTGTCGACTTCCAGCAAAGGATCGAACTGG
CBU0533-KO-F	ATGGAACTTATTAAAAGCGGC
CBU0533-KO-R	TCACCCTTCGGGGTTAGCG
CBU0533-comp-F	TTACTCAATGGAATTCGGCATTGTCGTCGGTACCCG
CBU0533-comp-R	GCTTCTCGAGGAATTCGGATTGGCAATTCTACAACAC
CBU0533-D156C-comp-F	GGTCTGGCCGGGGGCGTAG
CBU0533-D156C-comp-R	GCCCCCGGCCAGACCACATTGACCGTCAATCATATTCATTGC
CBU0533-T138M-comp-F	ATGGTAATTGTGGTTTTGGCTAACATC
CBU0533-T138M-comp-R	AACCACAATTACCATTATTGGAATAGCCCACAAG
1169P-lysCA-NdeI-F	GCTCGCGTCGCATATGGAGCTCGGTACCCGGGGATCC
1169P-lysCA-NdeI-R	CATGCGCACCCATATGGATTAATTAGAGAACCTGTTTGTCGAC
CBU1657-5′-F	CGGTACCCGGGGATCCTGACCGCACATAAGAGGTTACTCGTTTTG
CBU1657-5′-R	CACCCATATGCGACGCGAGCGTCGAGAAATTTGTCTTACTCATCTTCGTTGTTC
CBU1657-3′-F	CGTCGCATATGGGTGCGCATGTACGTCTTCGCTCCTACCAGGCAATCGGACC
CBU1657-3′-R	GAACCTGTTTGTCGACCCTTTTAAGCGCCTTACCGAATTG
CBU1657-KO-F	ATGAGTAAATTATCCGTTTATATCATC
CBU1657-KO-R	TTAGATCCGTTGACATCGCTCTTGC
CBU1657-comp-F	TTACTCAATGGAATTCTATTTATCGGCGGCATTGGCCTTTTAG
CBU1657-comp-R	GCTTCTCGAGGAATTCGGTACGAATAGATCTTTTCAAAGGCAG
CBU0839-5′-F	CGGTACCCGGGGATCCGCGATATTGTCATCCGAATGTTC
CBU0839-5′-R	CACCCATATGCGACGCGAGCGTCGAGCTAGATCGAAAAACACTAACTAC
CBU0839-3′-F	CGTCGCATATGGGTGCGCATGTACGTCCAACCTCAGCAATTAGCAGAAGC
CBU0839-3′-R	GAACCTGTTTGTCGACCGCATGGGTAAAGACCAAATG
CBU0839-KO-F	ATGATGAATATGTTCTTGAG
CBU0839-KO-R	TTTAGGCGGCACGACGAATC
CBU0839-comp-F	TTACTCAATGGAATTCGTACCTGTGGAAGACGTTGAAAC
CBU0839-comp-R	GCTTCTCGAGGAATTCGGATGCCATTGTAAAGTTCATTACC
Primers for complementation of California 16 RSA350 and M44 Clone 1 RSA461	
CBU0845-comp-F	TTACTCAATGGAATTCGGTAAGCTGTTCTCCGAGCG
CBU0845-comp-R	GCTTCTCGAGGAATTCGCAAGGTCTAATTTATGAAAAC
Primers for complementation of Australia RSA297	
CBU1657-Aucomp-F	AGATTACGCTGTCGACTATTTATCGGCGGCATTGGCCTTTTAG
CBU1657-Aucomp-R	GCATGCCTCAGTCGACGGTACGAATAGATCTTTTCAAAGGCAG
