# Supplementary material for: Genetic mechanisms of Coxiella burnetii lipopolysaccharide phase variation
Source: PLoS Pathog. 2018 Feb 26;14(3):e1006922. doi: 10.1371/journal.ppat.1006922 (PMC5843353; doi:10.1371/journal.ppat.1006922)
Supplement: S6 Table — (PDF) [file ppat.1006922.s011.pdf]

S6 Table. Construction of plasmids used in this study

Construction of pJC-Kan

The kanamycin gene was amplified from pJB-Kan by PCR using Kan-forpJC-F and Kan-forpJC-R oligonucleotides. The *kan* PCR product was cloned by In-Fusion into ApaI/NheI-digested pJC-CAT to create pJC-Kan.

Construction of pCR-BluntII::*1169^P*-CAT

A gBlock (Integrated DNA technologies) DNA fragment containing a *loxP* flanked *cbu1169* promoter-controlled chloramphenicol gene upstream of an *E. coli* terminator (*loxP*-*1169^P*-CAT-term-*loxP*) was cloned into pCR-BluntII-topo using TOPO PCR cloning technology (ThermoFisher Scientific) to create pCR-BluntII::*1169^P*-CAT.

Construction of pJC-Kan::*cbu0678tr*-5′3′-CAT

The 5′ and 3′ regions of *cbu0678*, which contain a truncated *cbu0678* sequence and upstream and downstream DNA, respectively, were amplified by PCR from Nine Mile RSA493 phase I (NMI) genomic DNA using the primer pairs CBU0678tr-5′-F/CBU0678tr-5′-R and CBU0678tr-3′-F/CBU0678tr-3′-R, respectively. The 5′ and 3′ PCR products were cloned into BamHI/SalI digested pJC-Kan by In-Fusion, resulting in formation of an internal NdeI site between the 5′ and 3′ fragments and creation of pJC-Kan::*cbu0678tr*-5′3′. The *1169^P*-CAT cassette was amplified from pJB-CAT by PCR with 1169P-CAT-NdeI-F and 1169P-CAT-NdeI-R primers and cloned by In-Fusion into NdeI-digested pJC-Kan::*cbu0678tr*-5′3′ to create pJC-Kan::*cbu0678tr*-5′3′-CAT.

Oligonucleotides	Sequence (5′ to 3′)
CBU0678tr-5'-F	CGGTACCCGGGGATCCTGTTTGAAAATGTTGATTATGTTTTCC
CBU0678tr-5'-R	CACCCATATGCGACGCGAGCGTCGAGAATGAATCGAGGGCTTCATGATCGC
CBU0678tr-3'-F	CGTCGCATATGGGTGCGCATGTACGTCCGATAAAAGAAAATGAGACGCTGGTAAC
CBU0678tr-3'-R	GAACCTGTTTGTCGACCACAGCAGGGTCATGCACCTGTATC
1169P-CAT-NdeI-F	GCTCGCGTCGCATATGATAACTTCGTATAGCATACATTATACGAAGTTATATGGCTTCGTTTCGCAGC
1169P-CAT-NdeI-R	CATGCGCACCCATATGATAACTTCGTATAATGTATGCTATACGAAGTTATTATAAACGCAGAAAGGCCCAC

Construction of pJC-Kan::*cbu1655*-5′3′-CAT

The 5′ and 3′ regions of *cbu1655* were amplified by PCR from Nine Mile RSA439 phase II (NMII) genomic DNA using the primer pairs CBU1655-5′-F/CBU1655-5′-R and CBU1655-3′-F/CBU1655-3′-R, respectively. The 5′ and 3′ PCR products were cloned into BamHI/SalI-digested

pJC-Kan by In-Fusion, resulting in formation of an internal NdeI site between the 5′ and 3′ fragments and creation of pJC-Kan::*cbu1655*-5′3′. The *1169*^{P}-CAT cassette was amplified from pCR-BluntII::*1169*^{P}-CAT by PCR with 1169P-CAT-NdeI-F and 1169P-CAT-NdeI-R primers and cloned by In-Fusion into NdeI-digested pJC-Kan::*cbu1655*-5′3′ to create pJC-Kan::*cbu1655*-5′3′-CAT.

Oligonucleotides	Sequence (5′ to 3′)
CBU1655-5'-F	CGGTACCCGGGGATCCCATTGATTTAATTTACCTCGATTGGC
CBU1655-5'-R	CACCCATATGCGACGCGAGCGTCGAGTCAAAGACTAAAGAGGCAGC
CBU1655-3'-F	CGTCGCATATGGGTGCGCATGTACGTCGCGGTTATCTTAATTTAATCTACTC
CBU1655-3'-R	GAACCTGTTTGTCGACAACATCAGCGTTGGCGGACATTG
1169P-CAT-NdeI-F	GCTCGCGTCGCATATGATAACTTCGTATAGCATACATTATACGAAGTTATAT GGCTTCGTTTCGCAGC
1169P-CAT-NdeI-R	CATGCGCACCCATATGATAACTTCGTATAATGTATGCTATACGAAGTTATTA TAAACGCAGAAAGGCCCAC

Construction of pJC-CAT::*1169*^{P}*-lysCA*

The CBU1169 promoter (*1169*^{P}) was amplified by PCR from pJB-CAT with 1169^P-forLysCA-F and 1169^P-forLysCA-R oligonucleotides. The *lysCA* fusion gene (*lpp01774*) was amplified by PCR from *Legionella pneumophila* JR32 genomic DNA with primers LysCA-F and LysCA-R. The *1169*^{P} and *lysCA* PCR products were cloned by In-Fusion into BamHI/SalI-digested pJC-CAT to create pJC-CAT-*1169*^{P}*-lysCA*.

Oligonucleotides	Sequence (5′ to 3′)
1169P-forLysCA-F	CGGTACCCGGGGATCCATAACTTCGTATAGCATACATTATACGAAGTTATAT GGCTTCGTTTCGCAGCGAACTTG
1169P-forLysCA-R	CCTCCTTCATGAAGG
LysCA-F	CTTCATGAAGGAGGCTGCAGTTGAGCGGAATACTAATCATGCAAC
LysCA-R	GAACCTGTTTGTCGACATAACTTCGTATAATGTATGCTATACGAAGTTATTTA TTCCAGAACTATTTCCTGAG

Construction of pJC-CAT::*cbu0533*-5′3′*-lysCA*

The 5′ and 3′ regions of *cbu0533* were amplified by PCR from NMI genomic DNA using the primer pairs CBU0533-5′-F/CBU0533-5′-R and CBU0533-3′-F/ CBU0533-3′-R, respectively. The 5′ and 3′ PCR products were cloned into BamHI/SalI-digested pJC-CAT by In-Fusion, resulting in formation of an internal NdeI site between the 5′ and 3′ fragments and creation of pJC-CAT::*cbu0533*-5′3′. The *1169*^{P}*-lysCA* cassette was amplified from pJC-CAT::*1169*^{P}*-lysCA* by PCR with 1169P-lysCA-NdeI-F and 1169P-lysCA-NdeI-R primers and cloned by In-Fusion into NdeI-digested pJC-CAT::*cbu0533*-5′3′ to create pJC-CAT::*cbu0533*-5′3′*-lysCA*.

Oligonucleotides	Sequence (5′ to 3′)
CBU0533-5'-F	CGGTACCCGGGGATCCCAGACCCAAAAGTTATTGTGGC
CBU0533-5'-R	CACCCATATGCGACGCGAGCGTCGAGAAAAAGTCACATCCTGCAGTTCT
CBU0533-3'-F	CGTCGCATATGGGTGCGCATGTACGTCGCTCTTGTGACTAAAACCTCC
CBU0533-3'-R	GAACCTGTTTGTCGACTTCCAGCAAAGGATCGAACTGG
1169P-lysCA-NdeI-F	GCTCGCGTCGCATATGGAGCTCGGTACCCGGGGATCC
1169P-lysCA-NdeI-R	CATGCGCACCCATATGGATTAATTAGAGAACCTGTTTGTCGAC

Construction of pJC-CAT::*cbu1657*-5′3′-*lysCA*

The 5′ and 3′ regions of *cbu1657* were amplified by PCR from NMII genomic DNA using the primer pairs CBU1657-5′-F/CBU1657-5′-R and CBU1657-3′-F/ CBU1657-3′-R, respectively. The 5′ and 3′ PCR products were cloned into BamHI/SalI-digested pJC-CAT by In-Fusion, resulting in formation of an internal NdeI site between the 5′ and 3′ fragments and creation of pJC-CAT::*cbu1657*-*5′*3′. The *1169*^{P}*-lysCA* cassette was amplified from pJC-CAT::*1169*^{P}*-lysCA* by PCR with 1169P-lysCA-NdeI-F and 1169P-lysCA-NdeI-R primers and cloned by In-Fusion into NdeI-digested pJC-CAT::*cbu1657*-*5′*3′ to create pJC-CAT::*cbu1657*-*5′*3′-*lysCA*.

Oligonucleotides	Sequence (5′ to 3′)
CBU1657-5'-F	CGGTACCCGGGGATCCTGACCGCACATAAGAGGTTACTCGTTTTG
CBU1657-5'-R	CACCCATATGCGACGCGAGCGTCGAGAAATTTGTCTTACTCATCTTCGTTGTTC
CBU1657-3'-F	CGTCGCATATGGGTGCGCATGTACGTCTTCGCTCCTACCAGGCAATCGGACC
CBU1657-3'-R	GAACCTGTTTGTCGACCCTTTTAAGCGCCTTACCGAATTG
1169P-lysCA-NdeI-F	GCTCGCGTCGCATATGGAGCTCGGTACCCGGGGATCC
1169P-lysCA-NdeI-R	CATGCGCACCCATATGGATTAATTAGAGAACCTGTTTGTCGAC

Construction of pJC-CAT::*cbu0839*-5′3′-*lysCA*

The 5′ and 3′ regions of *cbu0839* were amplified by PCR from NMI genomic DNA using the primer pairs CBU0839-5′-F/CBU0839-5′-R and CBU0839-3′-F/ CBU839-3′-R, respectively. The 5′ and 3′ PCR products were cloned into BamHI/SalI-digested pJC-CAT by In-Fusion, resulting in formation of an internal NdeI site between the 5′ and 3′ fragments and creation of pJC-CAT::*cbu0839*-*5′*3′. The *1169*^{P}*-lysCA* cassette was amplified from pJC-CAT::*1169*^{P}*-lysCA* by PCR with 1169P-lysCA-NdeI-F and 1169P-lysCA-NdeI-R primers and cloned by In-Fusion into NdeI-digested pJC-CAT::*cbu0839*-*5′*3′ to create pJC-CAT::*cbu0839*-*5′*3′-*lysCA*.

Oligonucleotides	Sequence (5′ to 3′)
CBU1657-5'-F	CGGTACCCGGGGATCCGCGATATTGTCATCCGAATGTTC

CBU1657-5'-R	CACCCATATGCGACGCGAGCGTCGAGCTAGATCGAAAAACACTAACTAC
CBU1657-3'-F	CGTCGCATATGGGTGCGCATGTACGTCCAACCTCAGCAATTAGCAGAAGC
CBU1657-3'-R	GAACCTGTTTGTCGACCGCATGGGTAAAGACCAAATG
1169P-lysCA-NdeI-F	GCTCGCGTCGCATATGGAGCTCGGTACCCGGGGATCC
1169P-lysCA-NdeI-R	CATGCGCACCCATATGGATTAATTAGAGAACCTGTTTGTCGAC

Construction of pJB-Kan::*cbu0678*comp-I

The *cbu0678* gene was amplified by PCR from NMI genomic DNA with CBU0678tr-comp-F and CBU0678tr-comp-R primers. The resulting fragment was cloned by In-Fusion into PstI-digested pJB-Kan-2xHA to create pJB-Kan::*cbu0678*comp-I.

Oligonucleotides	Sequence (5′ to 3′)
CBU0678tr-comp-F	CTTCATGAAGGAGGCTGCAGATGTTGCTGAAACGATACCGTC
CBU0678tr-comp-R	TCGTATGGGTACATCTGCAGATAGCGAATGACTTCATATTTAGG

Construction of pJB-Kan::*cbu0678*comp-P74A

The N-terminus of *cbu0678*, containing a mutation resulting in a P74A amino acid change, and the *cbu0678* C-terminus, were amplified by PCR from NMI genomic DNA with CBU0678tr-comp-F/CBU0678-P74A-comp-R and CBU0678-P74A-comp-F/CBU0678tr-comp-R primer pairs. The resulting fragments were cloned by In-Fusion into PstI-digested pJB-Kan-2xHA to create pJB-Kan::*cbu0678*comp-P74A.

Oligonucleotides	Sequence (5′ to 3′)
CBU0678tr-comp-F	CTTCATGAAGGAGGCTGCAGATGTTGCTGAAACGATACCGTC
CBU0678-P74A-comp-F	GCTCACATTCCGCAAGATTTGCG
CBU0678-P74A-comp-R	TTGCGGAATGTGAGCACGATAATGCCCCTTAACAATG
CBU0678tr-comp-R	TCGTATGGGTACATCTGCAGATAGCGAATGACTTCATATTTAGG

Construction of pJB-Kan::*cbu0678*comp-G369R

The N-terminus of *cbu0678*, containing a mutation resulting in a G369R amino acid change, and the *cbu0678* C-terminus, were amplified by PCR from NMI genomic DNA with CBU0678tr-comp-F/CBU0678-G369R-comp-R and CBU0678-G369R-comp-F/CBU0678tr-comp-R primer pairs. The resulting fragments were cloned by In-Fusion into PstI-digested pJB-Kan-2xHA to create pJB-Kan::*cbu0678*comp-G369R.

Oligonucleotides	Sequence (5′ to 3′)
CBU0678tr-comp-F	CTTCATGAAGGAGGCTGCAGATGTTGCTGAAACGATACCGTC
CBU0678-G369-comp-F	AGAAATATAACCGAATTTAGAAATTTCG
CBU0678-G369R-comp-R	TTCGGTTATATTTCTCCACCGACTTGCCACCTGAC
CBU0678tr-comp-R	TCGTATGGGTACATCTGCAGATAGCGAATGACTTCATATTTAGG

Construction of pJB-Kan::*cbu0678*comp-P74A/G369R

The N-terminus of *cbu0678* containing a mutation resulting in a P74A amino acid change, the central region of *cbu0678* containing a mutation resulting in a G369R amino acid change, and the *cbu0678* C-terminus were amplified by PCR from NMI genomic DNA with CBU0678tr-comp-F/CBU0678-P74A-comp-R, CBU0678-P74A-comp-F/CBU0678-G369R-comp-R and CBU0678-G369R-comp-F/CBU0678tr-comp-R primer pairs, repsectively. The resulting 3 fragments were cloned by In-Fusion into PstI-digested pJB-Kan-2xHA to create pJB-Kan::*cbu0678*comp-P74A/G369R.

Oligonucleotides	Sequence (5′ to 3′)
CBU0678tr-comp-F	CTTCATGAAGGAGGCTGCAGATGTTGCTGAAACGATACCGTC
CBU0678-P74A-comp-F	GCTCACATTCCGCAAGATTTGCG
CBU0678-P74A-comp-R	TTGCGGAATGTGAGCACGATAATGCCCCTTAACAATG
CBU0678-G369-comp-F	AGAAATATAACCGAATTTAGAAATTTCG
CBU0678-G369R-comp-R	TTCGGTTATATTTCTCCACCGACTTGCCACCTGAC
CBU0678tr-comp-R	TCGTATGGGTACATCTGCAGATAGCGAATGACTTCATATTTAGG

Construction of pJB-CAT::*cbu1657*comp-II

The *cbu1657* promoter (*cbu1657*^{P}), along with *cbu1657*, were amplified by PCR from NMII genomic DNA using CBU1657-Aucomp-F and CBU1657-Aucomp-R primers. The corresponding PCR product was cloned by In-Fusion into EcoRI/SalI-digested pJB-CAT-2xHA to create pJB-CAT::*cbu1657*comp-II.

Oligonucleotides	Sequence (5′ to 3′)
CBU1657-Aucomp-F	ACAGGAAACAGAATTCTCAGCGAATACTTCGAAAGAGATAG
CBU1657-Aucomp-R	CGGTACGAATAGATCTTTACTTGAAATTTTGTGTACCGGC

Construction of pMiniTn7T-Kan

The *1169*^{P}*-Kan* cassette was amplified by PCR from pJB-Kan with 1169P-Kan-forTn7T-F and 1169P-Kan-forTn7T-R primers. The resulting fragment (*1169*^{P}*-Kan*) was cloned by In-Fusion into EcoRI-digested pMiniTn7T-CAT to create pMiniTn7T-Kan.

Oligonucleotides	Sequence (5′ to 3′)
1169P-Kan-forTn7T-F	TATCGATACCGTCGACATGGCTTCGTTTCGCAGCGAAC
1169P-Kan-forTn7T-R	GGGGTTCGAGGTCGACTTATCAGAAGAACTCGTCAAGAAGG

Construction of pMiniTn7T-Kan::*cbu1655*comp-II

The *cbu1655* promoter (*cbu1655*^{P}), along with *cbu1655* were amplified by PCR from NMII gDNA with CBU1655-comp-F and CBU1655-comp-R primers. The resulting fragment (*cbu1655*^{P}*-cbu1655*) was cloned by In-Fusion into EcoRI-digested pMiniTn7T-Kan to create pMiniTn7T-Kan::*cbu1655*comp-II.

Oligonucleotides	Sequence (5′ to 3′)
CBU0678tr-comp-F	ACAGGAAACAGAATTCTCAGCGAATACTTCGAAAGAGATAG
CBU0678tr-comp-R	TCGTATGGGTACATCTGCAGATAGCGAATGACTTCATATTTAGG

Construction of pMiniTn7T-CAT::*cbu0533*comp-I and pMiniTn7T-CAT::*cbu0533*comp-II

The *cbu0533* promoter (*cbu0533*^{P}), along with *cbu0533-I* and *cbu0533-II*, were amplified by PCR from NMI and NMII genomic DNA, respectively, with CBU0533-comp-F and CBU0533-comp-R primers. The resulting fragments *cbu0533*^{P}*-cbu0533-I* and *cbu0533*^{P}*-cbu0533-II* were cloned by In-Fusion into EcoRI-digested pMiniTn7T-CAT to create pMiniTn7T-CAT::*cbu0533*comp-I and pMiniTn7T-CAT::*cbu0533*comp-II, respectively.

Oligonucleotides	Sequence (5′ to 3′)
CBU0533-comp-F	TTACTCAATGGAATTCGGCATTGTCGTCGGTACCCG
CBU0533-comp-R	GCTTCTCGAGGAATTCGGATTGGCAATTCTACAACAC

Construction of pMiniTn7T-CAT::*cbu0533*comp-D156C

The *cbu0533* promoter (*cbu0533^P*), along with the N-terminus of *cbu0533*, containing a mutation resulting in a D156C amino acid change, and the C-terminus of *cbu0533*, were amplified by PCR from NMI genomic DNA, with CBU0533-comp-F/CBU0533-D156C-comp-R and CBU0533-D156C-comp-F/CBU0533-comp-R primer pairs, respectively. The resulting fragments *cbu0533^P-cbu0533-D156C-N* and *cbu0533-D156C-C* were cloned by In-Fusion into EcoRI-digested pMiniTn7T-CAT to create pMiniTn7T-CAT::*cbu0533*comp-D156C, respectively.

Oligonucleotides	Sequence (5′ to 3′)
CBU0533-comp-F	TTACTCAATGGAATTCGGCATTGTCGTCGGTACCCG
CBU0533-D156C-comp-F	GGTCTGGCCGGGGGCGTAG
CBU0533-D156C-comp-R	GCCCCCGGCCAGACCACATTGACCGTCAATCATATTCATTGC
CBU0533-comp-R	GCTTCTCGAGGAATTCGGATTGGCAATTCTACAACAC

Construction of pMiniTn7T-CAT::*cbu0533*comp-T138M

The *cbu0533* promoter (*cbu0533^P*), along with the N-terminus of *cbu0533*, containing a mutation resulting in a T138M amino acid change, and the C-terminus of *cbu0533* were amplified by PCR from NMI genomic DNA, with CBU0533-comp-F/CBU0533-T138M-comp-R and CBU0533-T138M-comp-F/CBU0533-comp-R primer pairs, respectively. The resulting fragments *cbu0533^P-cbu0533-T138M-N* and *cbu0533-T138M-C* were cloned by In-Fusion into EcoRI-digested pMiniTn7T-CAT to create pMiniTn7T-CAT::*cbu0533-T138M-comp*, respectively.

Oligonucleotides	Sequence (5′ to 3′)
CBU0533-comp-F	TTACTCAATGGAATTCGGCATTGTCGTCGGTACCCG
CBU0533-T138M-comp-F	ATGGTAATTGTGGTTTTGGCTAACATC
CBU0533-T138M-comp-R	AACCACAATTACCATTATTGGAATAGCCCACAAG
CBU0533-comp-R	GCTTCTCGAGGAATTCGGATTGGCAATTCTACAACAC

Construction of pMiniTn7T-CAT::*cbu0845*comp-I

The *cbu0845* promoter (*cbu0845^P*), along with *cbu0845*, were amplified by PCR from NMI genomic DNA with CBU0845-comp-F and CBU0845-comp-R primers. The resulting fragment (*cbu0845^P-cbu0845*) was cloned by In-Fusion into EcoRI-digested pMiniTn7T-CAT to create pMiniTn7T-CAT::*cbu0845*comp-I.

Oligonucleotides	Sequence (5′ to 3′)
CBU0845-comp-F	ACAGGAAACAGAATTCTCAGCGAATACTTCGAAAGAGATAG

CBU0845-comp-R	TCGTATGGGTACATCTGCAGATAGCGAATGACTTCATATTTAGG

Construction of pMiniTn7T-CAT::*cbu1657*comp-II

The *cbu1657* promoter (*cbu1657*^{P}), along with *cbu1657*, were amplified by PCR from NMII genomic DNA with CBU1657-comp-F and CBU1657-comp-R primers. The resulting fragment (*cbu1657*^{P}-*cbu1657*) was cloned by In-Fusion into EcoRI-digested pMiniTn7T-CAT to create pMiniTn7T-CAT::*cbu1657comp*.

Oligonucleotides	Sequence (5′ to 3′)
CBU1657-comp-F	TTACTCAATGGAATTCTATTTATCGGCGGCATTGGCCTTTTAG
CBU1657-comp-R	GCTTCTCGAGGAATTCGGTACGAATAGATCTTTTCAAAGGCAG

Construction of pMiniTn7T-CAT::*cbu0839*comp-I

The *cbu0839* promoter (*cbu0839*^{P}), along with *cbu0839*, *cbu0840*, and *cbu0841* were amplified by PCR from NMI genomic DNA with CBU0839-comp-F and CBU0839-comp-R primers. The resulting fragment (*cbu0839*^{P}-*cbu0839-cbu0840-cbu0841*) was cloned by In-Fusion into EcoRI-digested pMiniTn7T-CAT to create pMiniTn7T-CAT::*cbu0839*comp-I.

Oligonucleotides	Sequence (5′ to 3′)
CBU0839-comp-F	TTACTCAATGGAATTCGTACCTGTGGAAGACGTTGAAAC
CBU0839-comp-R	GCTTCTCGAGGAATTCGGATGCCATTGTAAAGTTCATTACC
